# Diagnostic Accuracy of Real Time Reverse Transcriptase Polymerase Chain Reaction for Chikungunya Virus Detection: A Systematic Review and Meta‐Analysis

**DOI:** 10.1111/tmi.70203

**Published:** 2026-07-15

**Authors:** Róger J. Costa, Cleyde S. C. Marconi, Gleice K. Jesus, Gabriel O. Franco, Abevailton M. Silva, Maria B. Arriaga, Gúbio C. Soares, Silvia I. Sardi, Eduardo M. Netto

**Affiliations:** ^1^ Postgraduate Program in Medicine and Health Federal University of Bahia Salvador Bahia Brazil; ^2^ Fundação José Silveira Salvador Bahia Brazil; ^3^ Fundação Bahiana de Infectologia Salvador Bahia Brazil; ^4^ Institute of Health Sciences ‐ Virology Laboratory, Federal University of Bahia Salvador Bahia Brazil

**Keywords:** Accuracy, CHIKV, DTA, Molecular Biology, RT‐qPCR

## Abstract

**Background:**

Chikungunya virus (CHIKV) is an arbovirus that causes an acute febrile illness with intense muscle and joint pain, with the possibility of progressing to a chronic or neurological stage. We conducted a systematic review and meta‐analysis with the aim of evaluating the accuracy of real‐time reverse transcriptase polymerase chain reaction (RT‐qPCR) protocols for detecting CHIKV during the acute phase of symptoms.

**Methods:**

We searched the literature until December 5, 2024, including English, Portuguese, and Spanish, with no time restriction in PubMed, LILACS, and Web of Science. Studies that evaluated RT‐qPCR protocols compared to reference techniques such as viral isolation or validated RT‐qPCRs were included in the study. We collected data on sensitivity, specificity, accuracy, study design and methodological characteristics of RT‐qPCR. Quadas‐2 tool was used to assess the risk of bias and applicability. Subsequently, a meta‐analysis and meta‐regression were performed.

**Results:**

Twenty‐eight protocols were included in this systematic review, 28 index tests were extracted and 24 were included in quantitative analysis. According to meta‐analysis, RT‐qPCR for CHIKV detection had a combined accuracy of 96% (95% CI: 94 to 99%) and a summary sensitivity and specificity of 99.7% and 99.7%, respectively; however, the results need to be interpreted considering the high heterogeneity (78.1%) and publication bias (*p* = 0.002).

**Conclusion:**

RT‐qPCR is a highly accurate technique for detecting the Chikungunya virus during the acute phase of symptoms. Despite the high heterogeneity observed, all subgroup analyses demonstrated high overall performance.

## 1 Introduction

Chikungunya fever is a disease caused by Chikungunya virus (CHIKV) that provides an acute febrile condition and rash, like other arboviruses, but which stands out for its intense joint pain, which can last for weeks and require analgesic medication, and can progress to a chronic state in 43% of cases [1, 2]. In 2024 global estimates of 450.000 cases and over 160 deaths were reported worldwide by the ECDC, particularly concentrated in the Americas and other tropical regions [3].

Viral isolation is widely regarded as the definitive method for identifying the CHIKV; however, this technique requires substantial infrastructure, proficient technical personnel, and considerable financial investment, which complicates its application for routine diagnostic purposes [4]. Serological tests can also be used to diagnose Chikungunya fever; however, cases of false‐negative results and cross‐reactivity with the O'nyong‐nyong, Mayaro, and Ross River viruses have been documented [5]. A recent meta‐analysis showed that antigen tests, also applicable to the acute phase of symptoms, have a sensitivity of 85.8 and specificity of 93 in rapid tests; an 82.2, and 95.1 for ELISA [6].

In this context, real‐time reverse transcription polymerase chain reaction (RT‐qPCR) has emerged as a superior tool for acute phase diagnosis. By transcribing viral RNA into cDNA and exponentially amplifying specific targets, it allows for high‐sensitivity quantification and accurate differential diagnosis between co‐circulating arboviruses. Accurate and early diagnosis of CHIKV is necessary to guide clinical management, strengthen epidemiological surveillance, and is essential in the differential diagnosis of severe neurological complications such as encephalitis and Guillain–Barré syndrome [7]. The aim of this systematic review and meta‐analysis was to evaluate the accuracy of RT‐qPCR for the identification of CHIKV during the acute phase of symptoms, providing an updated evidence base for clinical and public health decision‐making.

## 2 Methods

### 2.1 Protocol and Registration

We conducted a systematic review and meta‐analysis according to the guidelines of the Preferred Reporting Items of Systematic reviews and Meta‐Analyses (PRISMA) [8] and used its specific extension for diagnostic accuracy studies. The systematic review was registered in the International Prospective Register of Systematic Reviews (PROSPERO) under registration number CRD42024508661.

### 2.2 Literature Search Strategy

The literature search was conducted in PubMed, Web of Science and LILACS databases up to December 2024, without time restrictions. Studies published in English, Spanish and Portuguese were considered eligible. This language restriction was applied considering that English is the predominant language in high‐impact scientific journal publications globally, including those from regions where CHIKV is endemic, and most of these publications are part of international databases exclusively encompassing those consulted in this review. The search strategy included the following descriptors: (‘Polymerase Chain Reaction’ [Mesh] or ‘Real Time Polymerase Chain Reaction’ [Mesh] or ‘Reverse Transcriptase Polymerase Chain Reaction’ [Mesh] or ‘Multiplex Polymerase Chain Reaction’ [Mesh]) and (‘Chikungunya Fever’ [Mesh] or ‘Chikungunya virus’ [Mesh]).

### 2.3 Inclusion and Exclusion Criteria

Studies were included if they: tested samples from patients with confirmed or suspected Chikungunya disease; evaluated the accuracy of the PCR technique; had RT‐PCR, RT‐qPCR, viral isolation, serology or other techniques as reference tests; contained sufficient data to make the 2 × 2 table. In addition, articles displaying conflicting study designs, such as reviews, meta‐analyses, letters to the editor, editorials, case reports; in silico protocols; articles using non‐human samples and articles lacking clinical samples pertaining to CHIKV or without a primary diagnostic focus were systematically excluded.

### 2.4 Study Selection

All records retrieved from the databases were imported into the Rayyan [9] reference manager (https://www.rayyan.ai/) for the screening process. After duplicate removal, three reviewers (RC, CM and GF) independently screened titles and abstracts according to predefined inclusion and exclusion criteria. Full‐text articles of potentially eligible studies were subsequently assessed. Disagreements at any stage of the screening process were initially resolved through discussion among the reviewers. When consensus could not be reached, a fourth reviewer (EN) acted as a judge and made the final decision based on the eligibility criteria. Reasons for inclusion or exclusion at the full‐text stage were systematically documented and reported in a PRISMA flow diagram.

### 2.5 Data Collection Process

Data extraction from eligible studies was performed independently by two authors (R.C. and C.M.) using a standardised extraction form in Microsoft Excel 365 with the following variables: Authors, year of publication, country of origin, study design, sample size, number of patients tested for CHIKV and other viruses, TP (true positive), FP (false positive), TN (true negative), FN (false negative), sensitivity, specificity, positive predictive value, negative predictive value, accuracy, comparison technique and type of detection of the index technique. To conclude this stage and reach a final consensus, the extracted data were compared, and any discrepancies discussed by the team; persistent conflicts were analysed and judged by a third reviewer (EN).

For studies in which sensitivity, specificity, positive predictive value, negative predictive value, and accuracy data were not reported, the measures were calculated using the Diagnostic test evaluation calculator in the MedCalc version 23.0.5.

### 2.6 Definitions for Data Extraction

For data collection, we classified two study designs, cross‐sectional and case–control. The cross‐sectional study design was defined as a participant with confirmed or suspected Chikungunya disease, while the case–control design included a sample of healthy patients in addition to a population of confirmed or suspected patients. For the meta‐analysis, other arboviruses and healthy patients were counted as negative cases.

### 2.7 Study Quality Assessment (Risk of Bias and Applicability)

The quality and risk of bias of the articles was assessed using the Quality Assessment of Diagnostic Accuracy Studies (QUADAS‐2) [10], a specific Cochrane tool for diagnostic accuracy studies, made up of four domains for risk of bias: Patient selection, index test, reference standard and flow and timing, and three domains for applicability. When one of the signalling questions was considered ‘unclear’ or ‘no’, the domain was assigned as ‘unclear’ or ‘high risk of bias’, respectively.

Graphs and tables for risk of bias and applicability were generated using Cochrane's Robvis tool (https://mcguinlu.shinyapps.io/robvis/). Three independent reviewers (R.C., C.M. and G.J.) assessed the quality and risk of bias in each study.

### 2.8 Statistical Analysis

The sensitivity, specificity and accuracy of each study were calculated with their respective confidence intervals in the case of studies with 2 × 2 tables; conversely, for studies that did not have 2 × 2 tables, the relevant data regarding true positives, true negatives, false positives and false negatives were extracted systematically based on the textual information provided. To assess the potential for publication bias, we used the funnel plot. The accuracy meta‐analysis was carried out using MedCalc version 23.0.5. In the process of determining whether to use the fixed or random effects model, the *I* ^2^ statistic was used [11], where a value greater than 75% denotes high heterogeneity, values ranging from 50% to 74% mean moderate heterogeneity, values between 25% and 49% indicate low heterogeneity and values close to 0% suggest no heterogeneity. The standard error was calculated using the accuracy confidence interval limits, according to the following formula: $\frac{\left(\text{upper limit}-\text{lower limit}\right)}{3,92}$ [12].

The HSROC curve was used to estimate the overall diagnostic performance of the assay, including pooled sensitivity and specificity, as well as to generate 95% confidence and 95% prediction regions. Confidence regions reflect the precision of the summary operating point, whereas prediction regions represent the expected range of diagnostic accuracy in future studies.

Between‐study heterogeneity was assessed within the HSROC framework by examining the dispersion of study estimates around the summary curve and by evaluating the width of the prediction region relative to the confidence region. Heterogeneity was interpreted qualitatively based on visual inspection of the HSROC plots and quantitatively through variance components derived from the hierarchical model.

To explore potential sources of heterogeneity, meta‐regression analyses were performed by incorporating study‐level covariates into the HSROC model. Pre‐specified covariates included study design (case–control versus cross‐sectional) and assay characteristics, such as assay format (singleplex vs. multiplex). These covariates were evaluated for their impact on diagnostic accuracy, primarily through changes in sensitivity and the position of the HSROC curve.

Model comparisons were conducted using likelihood ratio tests to assess whether the inclusion of covariates significantly improved model fit. Subgroup‐specific HSROC curves and summary estimates were generated to facilitate interpretation of covariate effects.

### 2.9 Sensitivity Analysis

Subgroup analysis for meta‐analysis was performed using the sensitivity and specificity of each study, and the SE was calculated based on the confidence interval of sensitivity or specificity. The analysis was grouped by detection method, SYBR Green or probe; type of reverse transcription, one‐step or two‐step; type of protocol, commercial or in‐house.

## 3 Results

### 3.1 Study Selection

The study selection process was conducted in accordance with PRISMA guidelines and is schematically presented in the flowchart shown in Figure 1. Following the stages of identification, screening and eligibility assessment, 26 studies were included in the systematic review, of which 22 were incorporated into the meta‐analysis and meta‐regression.

**FIGURE 1:**
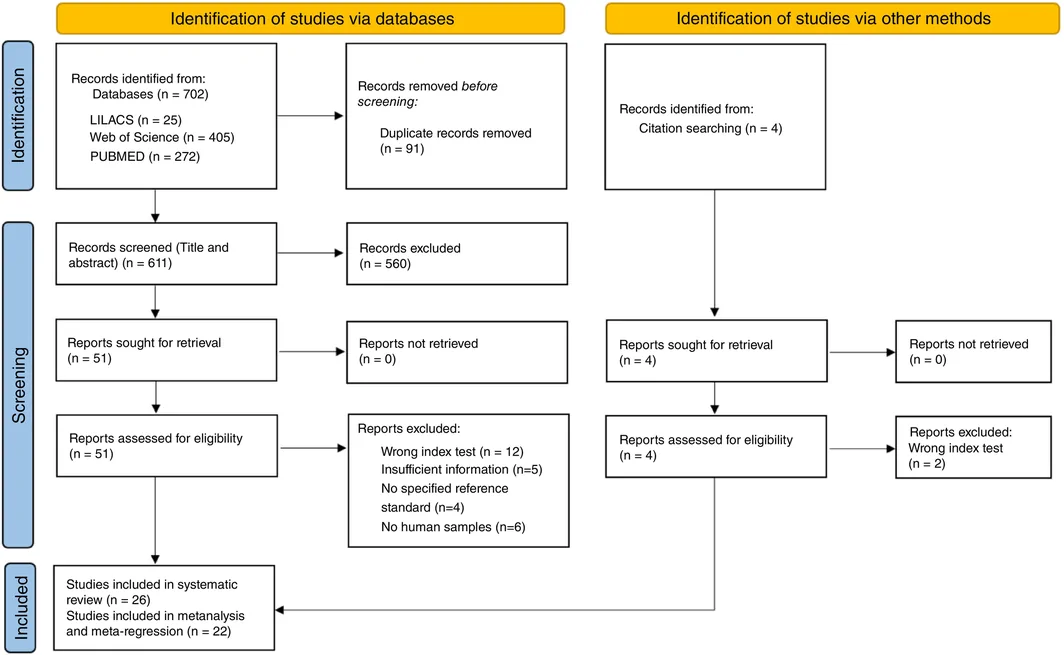
PRISMA flow diagram. Description of the selection flow of studies included in the systematic review and meta‐analysis.

### 3.2 Study Characteristics

Two studies [13, 14] which evaluated more than one protocol, each protocol by the same author, were identified as (1) and (2); two studies with the same author and year were also identified as (1) [15, 16] and (2) for differentiation. Three studies [17, 18, 19] did not have data for calculating accuracy because they only had data classified as true positives. One study [20] was not included in the meta‐analysis because it was classified as high risk of bias in the QUADAS‐2 evaluation. The technical and methodological characteristics of the protocols included are reported in Table 1. The map showing the origin of the specimens used in the protocols is available in the Supporting Information (Figure S1).

**TABLE 1:** General and technical characteristics of included protocols.

References	Study design	Protocol type	Sample size	Reference standard	Reverse transcription	Targets detection	Fluorescence method	Application	Sample type
Ali et al. [21]	Control–case	In‐house	17	RT‐qPCR	One‐step	Singleplex	Sybr green	Detection‐quantification	Serum
Andrew et al. (1) [13]	Cross‐sectional	In‐house	48	RT‐PCR and virus isolation	One‐step	Singleplex	Probe	Detection	Serum
Andrew et al. (2) [13]	Cross‐sectional	Commercial	33	RT‐PCR and virus isolation	One‐step	Singleplex	Probe	Detection	Serum
Boga et al. [17]	Cross‐sectional	In‐house	6	RT‐PCR	One‐step	Multiplex and singleplex	Probe	Detection	Whole blood
Carletti et al. [22]	Control–case	In‐house	24	RT‐PCR	One‐step	Singleplex	Probe	Detection‐quantification	Serum
Cecilia et al. [23]	Cross‐sectional	In‐house	94	RT‐PCR	One‐step	Multiplex	Probe	Detection‐quantification	Serum
Chen et al. [24]	Control–case	In‐house	77	RT‐PCR	One‐step	Multiplex	Sybr green	Detection‐quantification	Serum
Chiam et al. (1) [14]	Control–case	In‐house	50	RT‐qPCR	Two‐step	Singleplex	Sybr green	Quantification	Serum
Chiam et al. (2) [14]	Control–case	In‐house	50	RT‐qPCR	Two‐step	Singleplex	Sybr green	Quantification	Serum
Dakhave et al. [25]	Cross‐sectional	Commercial	60	RT‐qPCR	One‐step	Multiplex	Probe	Detection	Plasma
Edwards et al. [26]	Cross‐sectional	In‐house	55	RT‐PCR	One‐step	Singleplex	Probe	Detection	Serum
Edwards et al. [27]	Cross‐sectional	In‐house	164	RT‐qPCR	One‐step	Singleplex	Probe	Detection	Serum
Lanciotti et al. [28]	Cross‐sectional	NI	12	Virus isolation	—	Singleplex	Probe	Quantification	Serum
Lim et al. [29]	Cross‐sectional	In‐house	9	RT‐qPCR	One‐step	Singleplex	Probe	Quantification	Serum
Liu et al. [30]	Cross‐sectional	In‐house	10	Virus isolation and RT‐qPCR	One‐step	Multiplex	Probe	Quantification	Serum
Mansuy et al. [18]	Cross‐sectional	In‐house	5	RT‐qPCR	One‐step	Multiplex	Probe	Detection	Whole blood, plasma, serum and urine
Mishra et al. [31]	Cross‐sectional	In‐house	241	RT‐qPCR	One‐step	Multiplex	Probe	Quantification	Serum, urine
Naze et al. [32]	Control–case	In‐house	80	RT‐qPCR	One‐step	Multiplex	Probe	Quantification	Plasma
Pabbaraju et al. [33]	Cross‐sectional	In‐house	15	RT‐qPCR	One‐step	Multiplex	Probe	Detection	Serum, plasma and CSF
Panning et al. [34]	Cross‐sectional	Commercial	57	RT‐qPCR	One‐step	Singleplex	Probe	Detection	Plasma
Pongsiri et al. [20]	Cross‐sectional	In‐house	287	RT‐PCR	One‐step	Multiplex	Probe	Quantification	Serum
Santiago et al. [35]	Cross‐sectional	In‐house	155	RT‐qPCR	One‐step	Multiplex	Probe	Quantification	Serum, whole blood, urine
Santhosh et al. [36]	Control–case	In‐house	61	RT‐PCR	One‐step	Singleplex	Sybr green	Quantification	Serum
Simmons et al. [37]	Cross‐sectional	In‐house	50	Virus isolation	One‐step	Multiplex	Probe	Quantification	Serum
Waggoner et al. (1) [15]	Cross‐sectional	In‐house	81	RT‐qPCR	One‐step	Multiplex	Probe	Detection	Serum
Waggoner et al. (2) [16]	Cross‐sectional	In‐house	221	RT‐qPCR	One‐step	Multiplex	Probe	Detection	Serum
Wesselman et al. [38]	Cross‐sectional	In‐house	47	RT‐qPCR	One‐step	Multiplex	Probe	Detection	Plasma
Xu et al. [19]	Cross‐sectional	In‐house	7	RT‐qPCR	One‐step	Multiplex	Probe	Detection	Serum, plasma and urine

*Note:* Methodological characteristics of the included studies.

Abbreviations: CSF, cerebrospinal fluid; NI, no information.

### 3.3 Study Quality Assessment

The results of the assessment of risk of bias and applicability using QUADAS‐2 are shown in Figure 2. The traffic light plot can be found in Supporting Information.

**FIGURE 2:**
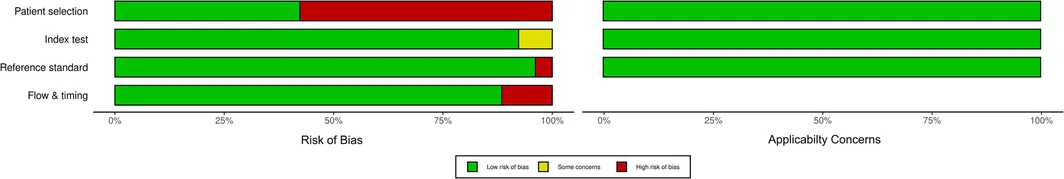
Overall percentage of risk of bias and applicability concerns using the QUADAS‐2 tool. Bar chart shows the percentage of studies classified by domain according to risk of bias and applicability.

Of the 26 studies included, 15 were classified as high risk of bias in patient selection. In the index test, 24 articles were classified as low risk and two as unclear risk. Twenty‐five articles were classified as low risk and one article was classified as high risk of bias in the reference technique domain and in the flow and follow‐up domain; 23 articles were classified as low risk and articles were classified as high risk. All the articles were classified as low risk of bias in the applicability domains.

We compared the accuracy of protocols with high risk and uncertain risk of bias to protocols with low risk of bias and found the same performance: 0.975 (95% CI: 0.934 to 1.000) vs. 0.961 (95% CI: 0.932 to 0.990), respectively.

### 3.4 Meta‐Analysis

The meta‐analysis of all included protocols' accuracy is reported in Figure 3. The analysis showed high heterogeneity (*I* ^2^ = 78%).

**FIGURE 3:**
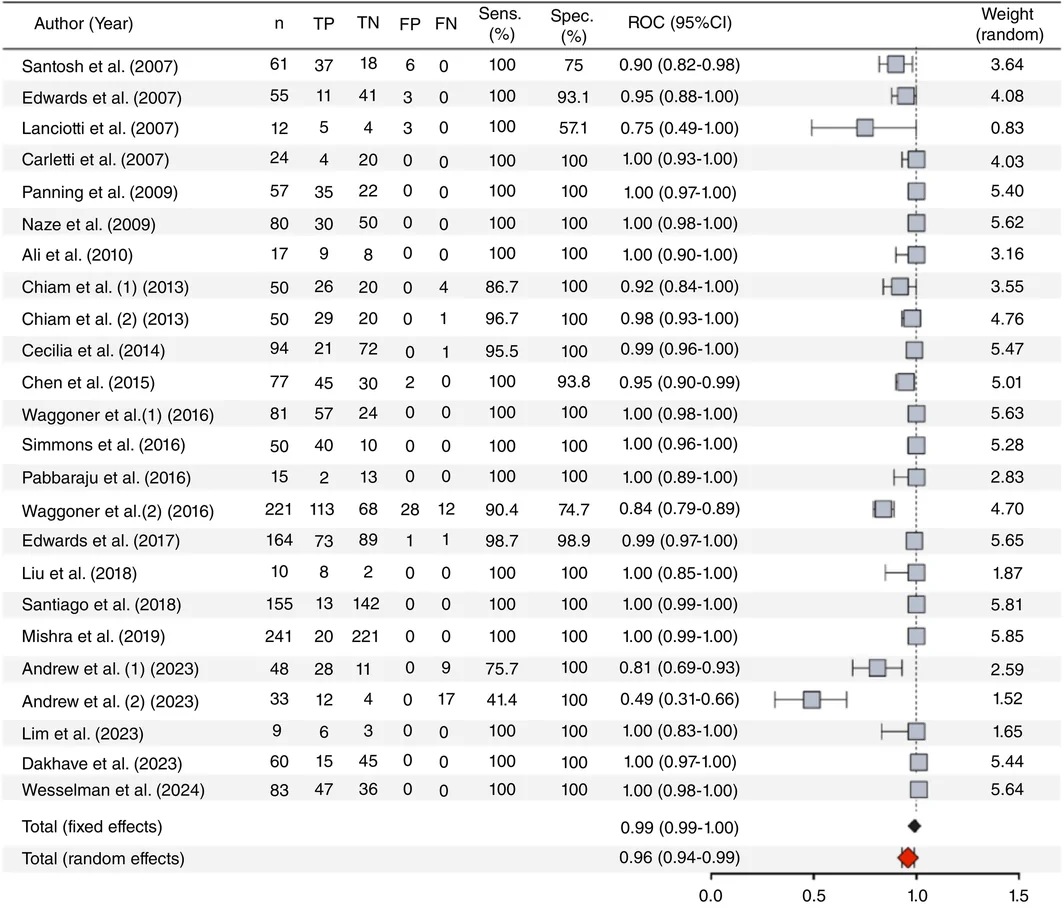
Forest plot reporting the accuracy of the studies included in the meta‐analysis. The squares represent the accuracy and the horizontal lines the 95% confidence interval of each study. TP, true positive; TN, true negative; FP, false positive; FN, false positive; CI, Confidence interval.

To assess the impact of protocols with a sample size < 30 participants, we performed a sensitivity analysis excluding these articles. No differences were found with respect to the main analysis (Figure S4).

The detection method (SYBR vs. probe), the type of protocol (commercial vs. in‐house), and the type of transcription (one‐step vs. two‐step) were also evaluated by sensitivity analysis (Table S1). No difference was observed between the detection method and the number of targets; the findings should be weighed according to the unequal number of samples between groups and the high heterogeneity.

### 3.5 Meta‐Regression

The HSROC model showed in Figure 4. Evidence of between‐study heterogeneity, predominantly affecting sensitivity. While specificity was consistently high across studies, sensitivity exhibited greater variability, as reflected by the width of the 95% prediction region. HSROC analyses were performed by study type (case‐control and cross sectional) and by assay type (multiplex and singleplex), as show in Figure 5.

**FIGURE 4:**
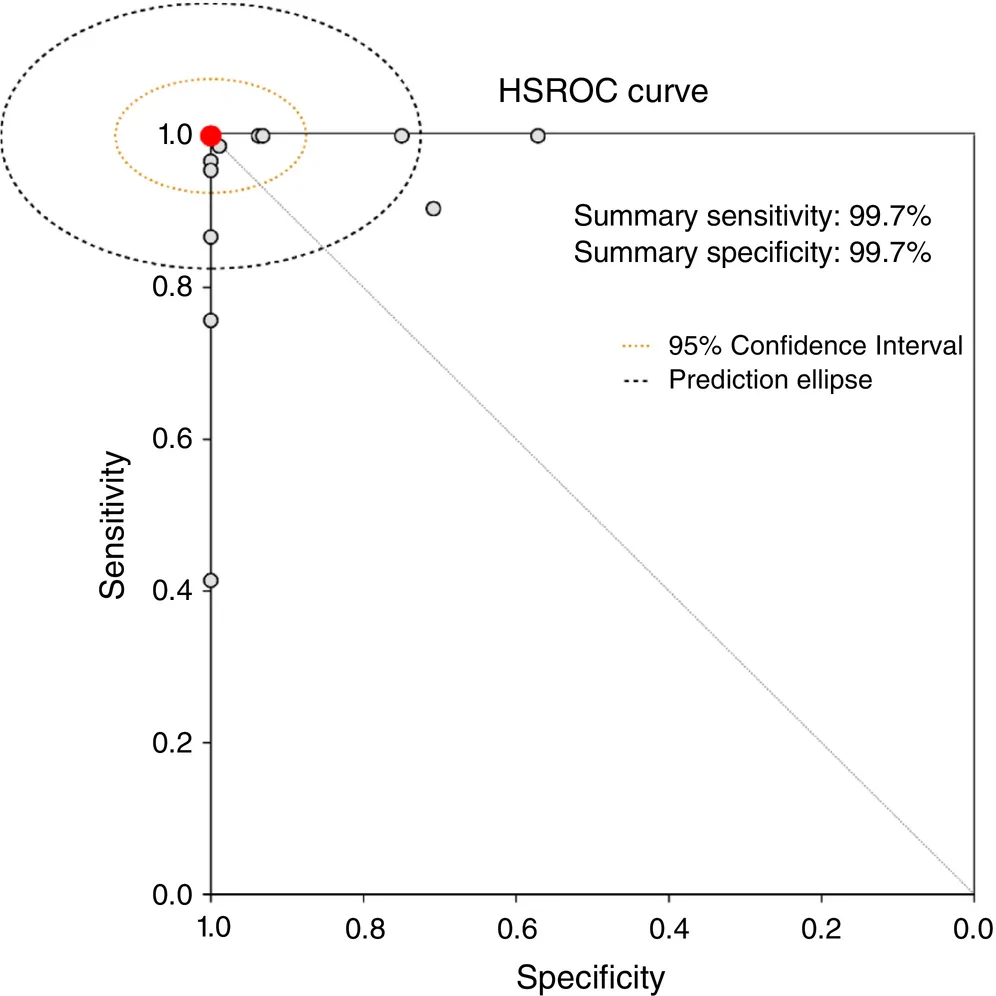
Hierarchical summary receiver operating characteristic (HSROC) curve. Grey circles represent individual studies. The red solid circle indicates the summary operating point, corresponding to the pooled sensitivity and specificity estimated by the hierarchical model. The solid line represents the HSROC curve. The yellow dashed ellipse denotes the 95% confidence region around the summary estimate, while the black dashed ellipse represents the 95% prediction region, reflecting between‐study heterogeneity.

**FIGURE 5:**
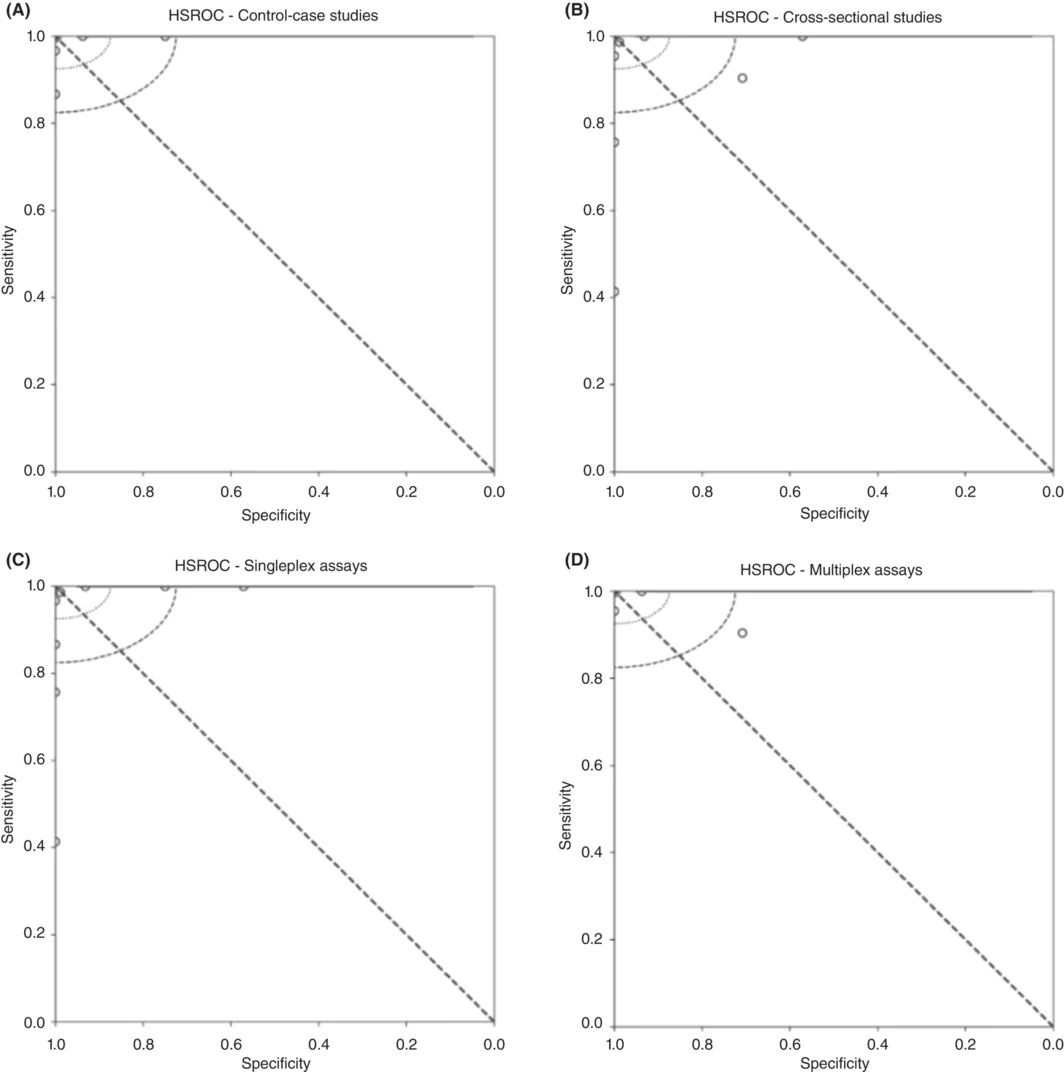
HSROC meta regression analysis by study‐level covariates. (A) Hierarchical Summary Receiver Operating Characteristic (HSROC) curves stratified by study design (case–control vs. cross‐sectional), (B) by assay format (singleplex vs. multiplex), (C) by protocol type (commercial vs. in‐house) and (D) by detection chemistry (SYBR Green vs. probe‐based assays). In each panel, open circles represent individual studies, solid circles indicate subgroup‐specific summary operating points and solid lines correspond to subgroup HSROC curves. Differences across panels illustrate the contribution of study design and assay characteristics to between‐study heterogeneity, primarily affecting sensitivity, while specificity remains consistently high across subgroups.

We observed high heterogeneity in the meta‐analysis, which was confirmed by meta‐regression. We also performed sensitivity analyses by removing protocols with high risk of bias, sample size < 30, convenience samples and study design, but none of the analyses determined the underlying cause of the high heterogeneity.

### 3.6 Publication Bias

The analysis revealed an asymmetric funnel plot, indicating publication bias (*p* = 0.002). The funnel plot is shown in Figure 6.

**FIGURE 6:**
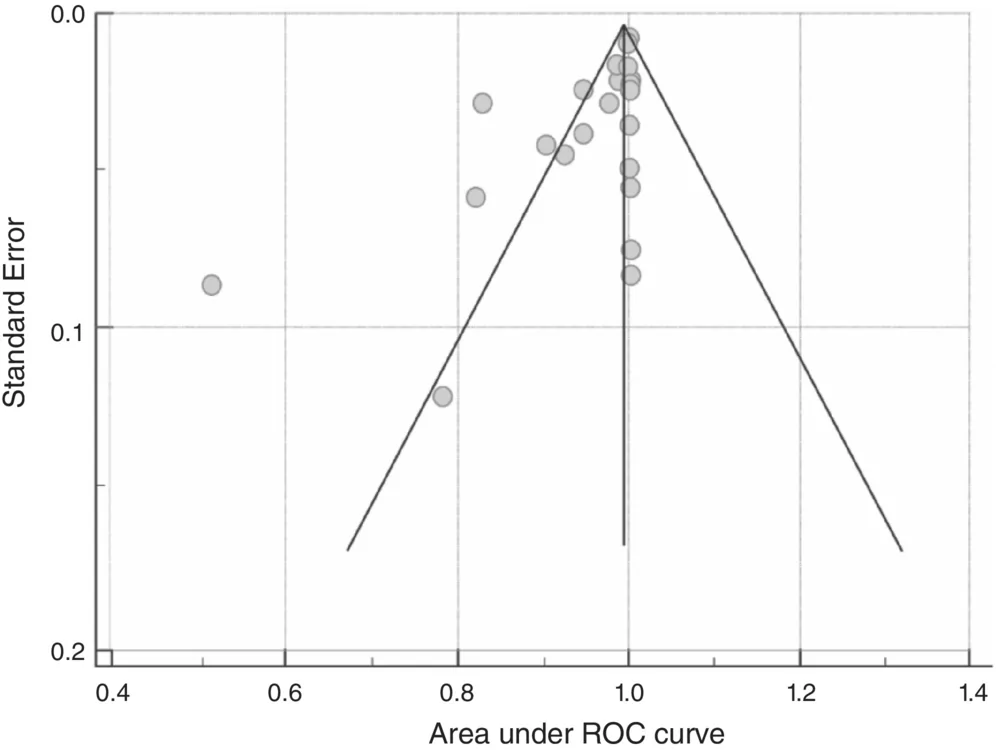
Funnel plot asymmetry test to assess publication bias. Each circle represents an individual study.

## 4 Discussion

Our meta‐analysis showed that RT‐qPCR has an accuracy of 96% (95% CI: 94% to 99%) in diagnosing CHIKV during the acute phase of symptoms. A previous meta‐analysis [39] analysed the diagnostic usefulness of the RT‐qPCR technique for CHIKV, including 8 articles, registering an accuracy of 99% (95% CI: 98% to 99%), equivalent to the value found in our study. Our article overcame some of the limitations of the previous study, used QUADAS‐2 as a tool for assessing quality and risk of bias, included more articles and addressed the impact of the technical characteristics of RT‐qPCR on diagnostic accuracy. This paper updates the literature and provides additional information about the technical characteristics of RT‐qPCR in the diagnosis of CHIKV, such as detection method, type of fluorescence used and number of targets used in the assay.

Our meta‐regression analysis concludes that cross‐sectional and case–control protocols presented equivalent results and high heterogeneity. It is known that protocols using consecutive or random samples are more recommended for calculating accuracy, while convenience samples overestimate sensitivity and specificity [10, 40]. Another way to overestimate metrics in a study of this type of design is characterised by protocols that used retrospective or archived samples. However, the analysis performed in our study, excluding protocols with convenience samples, did not confirm this.

Regarding methodological quality and risk of bias, the QUADAS‐2 tool, in the patient selection domain, classifies studies that use convenience samples or samples composed of healthy individuals as having a high risk of bias because these criteria tend to overestimate the study's performance measures. The inclusion of convenience samples using archived or biobank specimens can be attributed to the low prevalence of CHIKV in the country that developed the technique. On the other hand, the inclusion of healthy patients in the study (case–control design) only overestimates specificity and does not benefit clinical validation. Despite statistical equivalence in the analyses performed, both introduce bias and should be avoided in future protocols.

The results of the meta‐analysis indicated the presence of publication bias, evidenced by funnel plot asymmetry and corroborated by Egger's and Begg's tests. This finding suggests that the distribution of studies was not random in relation to the effect estimates, potentially reflecting distortions in the available literature. A plausible explanation is the underrepresentation of data showing performance below that expected for RT‐qPCR assays, since protocols with sensitivity and specificity estimates lower than commonly reported standards are less likely to be submitted or accepted for publication. In addition, language bias may have contributed to the observed asymmetry, as endemic countries for Chikungunya disease, such as India and Malaysia, may have published protocols in national journals or in local languages that were not covered by the search strategy adopted in this review. Collectively, these factors may have led to an overestimation of diagnostic accuracy measures in quantitative synthesis.

Qualitative analysis of the included studies demonstrated a progressive change in trial design after 2014, with a consistent trend towards incorporating viral targets other than CHIKV in RT‐qPCR. From an analytical standpoint, well‐validated multiplex assays maintain performance comparable to singleplex tests. From a clinical utility perspective, the expansion of the molecular panel increases the overall diagnostic value of the technique, especially in endemic regions where CHIKV, DENV and ZIKV share vectors, geographic distribution and similar initial clinical manifestations. In quantitative analysis, multiplex and singleplex assays performed equivalently [41].

We analysed methodological characteristics that could interfere with the protocols, such as the type of detection (probe versus SYBR), type of protocol (commercial versus in‐house) and type of transcription (one‐step versus two‐step). The analysis demonstrated equivalence between the groups, but the difference in sample size in the comparisons is a strong limitation for data interpretation.

Only one study with two protocols showed lower accuracy than the others. This can be attributed to the storage of specimens at −20°C, which is unsuitable for RNA viruses over long periods. However, as this finding is limited to a single investigation, caution is required when generalising this premise, and it must be validated in independent studies.

One of the main strengths of this review is the robust quantitative approach, including meta‐analysis, meta‐regression and sensitivity analyses, which demonstrated high estimates in univariate and bivariate models. In addition, we performed a comprehensive assessment of quality and risk of bias.

However, the estimates generated should be interpreted considering several significant limitations. Most notably, the high statistical heterogeneity among the included studies remained unexplained despite exploratory analyses. Furthermore, the presence of publication bias implies a possible overestimation of performance. Finally, the language restriction applied during study selection constitutes a potential source of bias, as relevant global scientific evidence may have been inadvertently excluded.

## 5 Conclusion

This systematic review provides a comprehensive assessment of the accuracy of RT‐qPCR for CHIKV detection. Meta‐analysis and meta‐regression demonstrated high accuracy, sensitivity and specificity. However, the high metrics should be evaluated in conjunction with the high heterogeneity identified in the meta‐analysis, which could not be explained by sensitivity analyses and meta‐regression. The findings of this review suggest that future validation and standardisation efforts for RT‐qPCR in Chikungunya diagnosis should prioritise methodological rigour in clinical study design. Prospective cross‐sectional studies with consecutive symptomatic patients and clearly defined reference standards are essential to ensure accurate case classification and reduce spectrum bias. Transparent reporting of patient selection criteria, timing of sample collection and clinical characteristics is also critical to enhance comparability across studies and to minimise heterogeneity in diagnostic performance estimates.

## Funding

R.C. (88887.91553/2023‐00) and G.F. (88887.960010/2024‐00) are recipients of a Coordenaçao de Aperfeiçoamento de Pessoal de Nivel Superior (CAPES); C.M. (140187/2021‐9) and G.J. (166623/2023‐7) are recipients of the National Council for Scientific and Technological Development (CNPq) scholarship; the project received funding from CNPq (404193/2019.6). The funders were not involved in the conceptualization, data extraction, analysis, preparation of the manuscript and the decision to publish.

## Ethics Statement

The authors have nothing to report.

## Consent

The authors have nothing to report.

## Conflicts of Interest

The authors declare no conflicts of interest.

## Supporting information


**Data S1:** tmi70203‐sup‐0001‐Supinfo1.xlsx.

**Data S2:** tmi70203‐sup‐0002‐Supinfo2.docx.

**Data S3:** tmi70203‐sup‐0003‐Supinfo3.docx.

**Data S4:** tmi70203‐sup‐0004‐Supinfo4.xlsx.

**Data S5:** tmi70203‐sup‐0005‐Supinfo5.docx.

**Figure S1:** Map showing the sample origin of the selected studies.
**Figure S2:** Sensitivity analysis to assess heterogeneity using studies with case–control design.
**Figure S3:** Sensitivity analysis to assess heterogeneity using studies with cross‐sectional design.
**Figure S4:** Sensitivity analysis to evaluate the performance of protocols without studies with sample size less than 30.
**Figure S5:** Sensitivity analysis to evaluate the performance of protocols with random or consecutive sampling.
**Table S1:** Subgroup analyses diagnostic sensitivity and specificity of RT‐qPCR.

**Data S6:** tmi70203‐sup‐0007‐Supinfo7.tif.

## Data Availability

The data that supports the findings of this study are available in the Supporting Information of this article.
