# Supplementary material for: Diagnostic Accuracy of Real Time Reverse Transcriptase Polymerase Chain Reaction for Chikungunya Virus Detection: A Systematic Review and Meta‐Analysis
Source: Trop Med Int Health. 2026 Jul 15;31(11):1537–47. doi: 10.1111/tmi.70203 (PMC13727711; doi:10.1111/tmi.70203)
Supplement: Supplementary file 2 [file TMI-31-1537-s003.docx]

**Abbreviations**

cDNA - complementary deoxyribonucleic acid

CHIKV – Chikungunya vírus

CI - Confidence interval

CSF - Cerebrospinal fluid

DTA – Diagnostic test accuracy

FN – False negative

FP – False positive

PRISMA

QUADAS-2 - Quality Assessment of Diagnostic Accuracy Studies

RT-qPCR – Real time reverse transcriptase polymerase chain reaction

TN - True negative

TP – True positive
