# Supplementary material for: Diagnostic Accuracy of Real Time Reverse Transcriptase Polymerase Chain Reaction for Chikungunya Virus Detection: A Systematic Review and Meta‐Analysis
Source: Trop Med Int Health. 2026 Jul 15;31(11):1537–47. doi: 10.1111/tmi.70203 (PMC13727711; doi:10.1111/tmi.70203)
Supplement: Supplementary file 3 [file TMI-31-1537-s006.docx]

**Search strategy (PubMed)**

("Polymerase Chain Reaction" [Mesh] or "Real Time Polymerase Chain Reaction" [Mesh] or "Reverse Transcriptase Polymerase Chain Reaction"[Mesh] or "Multiplex Polymerase Chain Reaction" [Mesh]) and ("Chikungunya Fever"[Mesh] or "Chikungunya virus"[Mesh])

**Search strategy (Web of Science)**

(ALL=(Polymerase Chain Reaction) OR ALL=(Real-Time Polymerase Chain Reaction) OR ALL=(Reverse Transcriptase Polymerase Chain Reaction) OR ALL=(Multiplex Polymerase Chain Reaction)) AND (ALL=(chikungunya fever) ALL=(chikungunya virus))

**Search strategy (LILACS)**

((reação em cadeia da polimerase) OR (reação em cadeia da polimerase em tempo real) OR (reação em cadeia da polimerase transcriptase reversa) OR (reação em cadeia da polimerase multiplex)) AND ((febre chikungunya) OR (vírus chikungunya)) AND fulltext:("1" OR "1") AND db:("LILACS") AND la:("en" OR "es" OR "pt") AND instance:"lilacsplus"
