# Supplementary material for: Diagnostic Accuracy of Real Time Reverse Transcriptase Polymerase Chain Reaction for Chikungunya Virus Detection: A Systematic Review and Meta‐Analysis
Source: Trop Med Int Health. 2026 Jul 15;31(11):1537–47. doi: 10.1111/tmi.70203 (PMC13727711; doi:10.1111/tmi.70203)
Supplement: Supplementary file 4 [file TMI-31-1537-s007.docx]

**Supplementary Figure 1 - Map showing the sample origin of the selected studies.**

The total number of specimens included in the protocols was 2016, obtained prospectively or retrospectively from tourists or residents of China, Comoros, Cuba, Ecuador, Grenada, India, Laos, Madagascar, Malaysia, Mauritius, Nicaragua, Pakistan, Puerto Rico, Reunion, Seychelles, Singapore or Thailand.

**Supplementary Figure 2.** Sensitivity analysis to assess heterogeneity using studies with case-control design

**Supplementary Figure 3.** Sensitivity analysis to assess heterogeneity using studies with cross-sectional design

**Supplementary Figure 4.** Sensitivity analysis to evaluate the performance of protocols without studies with sample size less than 30.

**Supplementary Figure 5.** Sensitivity analysis to evaluate the performance of protocols with random or consecutive sampling.

**Supplementary Table 1. Subgroup analyses diagnostic sensitivity and specificity of RT-qPCR**

| **Subgroup** | **Number of protocols** | **Sensitivity (95% CI)** | **Specificity (95% CI)** |
| --- | --- | --- | --- |
| **Protocol Type** | | | |
| In-house | 20 | 98% (97 to 100) | 98% (95 to 100) |
| Commercial | 3 | 82% (52 to 100) | 100% (97 to 100) |
| **Fluorescence method** | | | |
| Probe | 19 | 96% (92 to 100) | 98% (96 to 100) |
| SYBR green | 5 | 99% (96 to 100) | 96% (89 to 100) |
| **Reverse Transcription** | | | |
| One-step | 21 | 98% (96 to 100) | 98% (95 to 100) |
| Two-step | 2 | 93% (84 to 100) | 100% (94 to 100) |

CI: Confidence interval

# Meta-analysis: Area under ROC curve – Chikungunya Accuracy (main analysis)

| Variable for studies | Author |
| --- | --- |
| Variable for Area under ROC curve (AUC) | Acuracia_Decimal |
| Variable for Standard Error of AUC | SE |

| Study | ROC Area | Standard Error | 95% CI | z | P | Weight (%) | |
| --- | --- | --- | --- | --- | --- | --- | --- |
|  |  |  |  |  |  | Fixed | Random |
| Ali et al. (2010) | 1,000 | 0,0498 | 0,902 to 1,000 |  |  | 0,27 | 3,16 |
| Andrew et al. (1) (2023) | 0,813 | 0,0604 | 0,694 to 0,931 |  |  | 0,18 | 2,59 |
| Andrew et al. (2) (2023) | 0,485 | 0,0910 | 0,306 to 0,663 |  |  | 0,080 | 1,52 |
| Carletti et al. (2007) | 1,000 | 0,0364 | 0,929 to 1,000 |  |  | 0,50 | 4,03 |
| Cecilia et al. (2014) | 0,989 | 0,0147 | 0,961 to 1,000 |  |  | 3,06 | 5,47 |
| Chen et al. (2015) | 0,947 | 0,0223 | 0,903 to 0,991 |  |  | 1,33 | 5,01 |
| Chiam et al. (1) (2013) | 0,920 | 0,0434 | 0,835 to 1,000 |  |  | 0,35 | 3,55 |
| Chiam et al. (2) (2013) | 0,980 | 0,0260 | 0,929 to 1,000 |  |  | 0,98 | 4,76 |
| Dakhave et al. (2023) | 1,000 | 0,0152 | 0,970 to 1,000 |  |  | 2,86 | 5,44 |
| Edwards et al. (2007) | 0,946 | 0,0357 | 0,876 to 1,000 |  |  | 0,52 | 4,08 |
| Edwards et al. (2017) | 0,988 | 0,0107 | 0,967 to 1,000 |  |  | 5,78 | 5,65 |
| Lanciotti et al. (2007) | 0,750 | 0,132 | 0,492 to 1,000 |  |  | 0,038 | 0,83 |
| Lim et al. (2023) | 1,000 | 0,0858 | 0,832 to 1,000 |  |  | 0,090 | 1,65 |
| Liu et al. (2018) | 1,000 | 0,0787 | 0,846 to 1,000 |  |  | 0,11 | 1,87 |
| Mishra et al. (2019) | 1,000 | 0,00388 | 0,992 to 1,000 |  |  | 43,93 | 5,85 |
| Naze et al. (2009) | 1,000 | 0,0115 | 0,977 to 1,000 |  |  | 4,99 | 5,62 |
| Pabbaraju et al. (2016) | 1,000 | 0,0556 | 0,891 to 1,000 |  |  | 0,21 | 2,83 |
| Panning et al. (2009) | 1,000 | 0,0160 | 0,969 to 1,000 |  |  | 2,58 | 5,40 |
| Santiago et al. (2018) | 1,000 | 0,00599 | 0,988 to 1,000 |  |  | 18,38 | 5,81 |
| Santosh et al. (2007) | 0,902 | 0,0421 | 0,819 to 0,984 |  |  | 0,37 | 3,64 |
| Simmons et al. (2016) | 1,000 | 0,0181 | 0,964 to 1,000 |  |  | 2,01 | 5,28 |
| Waggoner et al. (1) (2016) | 1,000 | 0,0114 | 0,978 to 1,000 |  |  | 5,13 | 5,63 |
| Waggoner et al. (2) (2016) | 0,819 | 0,0269 | 0,766 to 0,872 |  |  | 0,91 | 4,70 |
| Wesselman et al. (2024) | 1,000 | 0,0111 | 0,978 to 1,000 |  |  | 5,36 | 5,64 |
| Total (fixed effects) | 0,995 | 0,00257 | 0,990 to 1,000 | 386,994 | <0,001 | 100,00 | 100,00 |
| Total (random effects) | 0,963 | 0,0130 | 0,938 to 0,989 | 74,030 | <0,001 | 100,00 | 100,00 |

## Test for heterogeneity

| Q | 105,2287 |
| --- | --- |
| DF | 23 |
| Significance level | P < 0,0001 |
| I^2^ (inconsistency) | 78,14% |
| 95% CI for I^2^ | 67,97 to 85,09 |

## Publication bias

| Egger's test | |
| --- | --- |
| Intercept | -1,7544 |
| 95% CI | -2,8180 to -0,6908 |
| Significance level | P = 0,0024 |
| Begg's test | |
| Kendall's Tau | -0,5290 |
| Significance level | P = 0,0003 |

Saturday, January 11, 2025 20:24 - MedCalc^®^ version 23.1.1

# Meta-analysis: Area under ROC curve – One-step Specificity

| Variable for studies | Author |
| --- | --- |
| Variable for Area under ROC curve (AUC) | Especificidade |
| Variable for Standard Error of AUC | SE_Especificidade SE Especificidade |

| Study | ROC Area | Standard Error | 95% CI | z | P | Weight (%) | |
| --- | --- | --- | --- | --- | --- | --- | --- |
|  |  |  |  |  |  | Fixed | Random |
| Ali et al. (2010) | 1,000 | 0,0942 | 0,815 to 1,000 |  |  | 0,11 | 1,67 |
| Andrew et al. (1) (2023) | 1,000 | 0,0727 | 0,857 to 1,000 |  |  | 0,19 | 2,50 |
| Andrew et al. (2) (2023) | 1,000 | 0,154 | 0,699 to 1,000 |  |  | 0,042 | 0,70 |
| Carletti et al. (2007) | 1,000 | 0,0430 | 0,916 to 1,000 |  |  | 0,54 | 4,80 |
| Cecilia et al. (2014) | 1,000 | 0,0127 | 0,975 to 1,000 |  |  | 6,11 | 8,72 |
| Chen et al. (2015) | 0,938 | 0,0511 | 0,837 to 1,000 |  |  | 0,38 | 3,98 |
| Dakhave et al. (2023) | 1,000 | 0,0201 | 0,961 to 1,000 |  |  | 2,46 | 7,81 |
| Edwards et al. (2007) | 0,932 | 0,0440 | 0,846 to 1,000 |  |  | 0,51 | 4,69 |
| Edwards et al. (2017) | 0,989 | 0,0153 | 0,959 to 1,000 |  |  | 4,21 | 8,42 |
| Lim et al. (2023) | 1,000 | 0,181 | 0,646 to 1,000 |  |  | 0,030 | 0,52 |
| Liu et al. (2018) | 1,000 | 0,215 | 0,579 to 1,000 |  |  | 0,021 | 0,37 |
| Mishra et al. (2019) | 1,000 | 0,00423 | 0,992 to 1,000 |  |  | 55,23 | 9,38 |
| Naze et al. (2009) | 1,000 | 0,0181 | 0,964 to 1,000 |  |  | 3,01 | 8,07 |
| Pabbaraju et al. (2016) | 1,000 | 0,0630 | 0,876 to 1,000 |  |  | 0,25 | 3,06 |
| Panning et al. (2009) | 1,000 | 0,0394 | 0,923 to 1,000 |  |  | 0,64 | 5,21 |
| Santiago et al. (2018) | 1,000 | 0,00653 | 0,987 to 1,000 |  |  | 23,22 | 9,26 |
| Santosh et al. (2007) | 0,750 | 0,0942 | 0,565 to 0,935 |  |  | 0,11 | 1,67 |
| Simmons et al. (2016) | 1,000 | 0,0787 | 0,846 to 1,000 |  |  | 0,16 | 2,22 |
| Waggoner et al. (1) (2016) | 1,000 | 0,0364 | 0,929 to 1,000 |  |  | 0,75 | 5,58 |
| Waggoner et al. (2) (2016) | 0,708 | 0,0485 | 0,613 to 0,803 |  |  | 0,42 | 4,23 |
| Wesselman et al. (2024) | 1,000 | 0,0248 | 0,951 to 1,000 |  |  | 1,60 | 7,14 |
| Total (fixed effects) | 0,997 | 0,00315 | 0,991 to 1,000 | 316,912 | <0,001 | 100,00 | 100,00 |
| Total (random effects) | 0,977 | 0,0134 | 0,951 to 1,000 | 72,892 | <0,001 | 100,00 | 100,00 |

## Test for heterogeneity

| Q | 46,9717 |
| --- | --- |
| DF | 20 |
| Significance level | P = 0,0006 |
| I^2^ (inconsistency) | 57,42% |
| 95% CI for I^2^ | 30,82 to 73,80 |

## Publication bias

| Egger's test | |
| --- | --- |
| Intercept | -0,7573 |
| 95% CI | -1,5937 to 0,07910 |
| Significance level | P = 0,0734 |
| Begg's test | |
| Kendall's Tau | -0,6158 |
| Significance level | P = 0,0001 |

Saturday, January 11, 2025 21:11 - MedCalc^®^ version 23.1.1

# Meta-analysis: Area under ROC curve – Two-step Specificity

| Variable for studies | Author |
| --- | --- |
| Variable for Area under ROC curve (AUC) | Especificidade |
| Variable for Standard Error of AUC | SE_Especificidade SE Especificidade |

| Study | ROC Area | Standard Error | 95% CI | z | P | Weight (%) | |
| --- | --- | --- | --- | --- | --- | --- | --- |
|  |  |  |  |  |  | Fixed | Random |
| Chiam et al. (1) (2013) | 1,000 | 0,0430 | 0,916 to 1,000 |  |  | 50,00 | 50,00 |
| Chiam et al. (2) (2013) | 1,000 | 0,0430 | 0,916 to 1,000 |  |  | 50,00 | 50,00 |
| Total (fixed effects) | 1,000 | 0,0304 | 0,940 to 1,000 | 32,917 | <0,001 | 100,00 | 100,00 |
| Total (random effects) | 1,000 | 0,0304 | 0,940 to 1,000 | 32,917 | <0,001 | 100,00 | 100,00 |

## Test for heterogeneity

| Q | 0,0000 |
| --- | --- |
| DF | 1 |
| Significance level | P = 1,0000 |
| I^2^ (inconsistency) | 0,00% |
| 95% CI for I^2^ | 0,00 to 0,00 |

Saturday, January 11, 2025 21:12 - MedCalc^®^ version 23.1.1

# Meta-analysis: Area under ROC curve – Commercial Specificity

| Variable for studies | Author |
| --- | --- |
| Variable for Area under ROC curve (AUC) | Especificidade |
| Variable for Standard Error of AUC | SE_Especificidade SE Especificidade |

| Study | ROC Area | Standard Error | 95% CI | z | P | Weight (%) | |
| --- | --- | --- | --- | --- | --- | --- | --- |
|  |  |  |  |  |  | Fixed | Random |
| Andrew et al. (2) (2023) | 1,000 | 0,154 | 0,699 to 1,000 |  |  | 1,34 | 1,34 |
| Dakhave et al. (2023) | 1,000 | 0,0201 | 0,961 to 1,000 |  |  | 78,32 | 78,32 |
| Panning et al. (2009) | 1,000 | 0,0394 | 0,923 to 1,000 |  |  | 20,35 | 20,35 |
| Total (fixed effects) | 1,000 | 0,0178 | 0,965 to 1,000 | 56,278 | <0,001 | 100,00 | 100,00 |
| Total (random effects) | 1,000 | 0,0178 | 0,965 to 1,000 | 56,278 | <0,001 | 100,00 | 100,00 |

## Test for heterogeneity

| Q | 0,0000 |
| --- | --- |
| DF | 2 |
| Significance level | P = 1,0000 |
| I^2^ (inconsistency) | 0,00% |
| 95% CI for I^2^ | 0,00 to 0,00 |

## Publication bias

| Egger's test | |
| --- | --- |
| Intercept | -4,737E-15 |
| 95% CI | -1,2177E-13 to 1,1229E-13 |
| Significance level | P = 0,6976 |
| Begg's test | |
| Kendall's Tau | ? |
| Significance level | P = 1,0000 |

Saturday, January 11, 2025 21:08 - MedCalc^®^ version 23.1.1

# Meta-analysis: Area under ROC curve – in-house Specificity

| Variable for studies | Author |
| --- | --- |
| Variable for Area under ROC curve (AUC) | Especificidade |
| Variable for Standard Error of AUC | SE_Especificidade SE Especificidade |

| Study | ROC Area | Standard Error | 95% CI | z | P | Weight (%) | |
| --- | --- | --- | --- | --- | --- | --- | --- |
|  |  |  |  |  |  | Fixed | Random |
| Ali et al. (2010) | 1,000 | 0,0942 | 0,815 to 1,000 |  |  | 0,11 | 1,86 |
| Andrew et al. (1) (2023) | 1,000 | 0,0727 | 0,857 to 1,000 |  |  | 0,19 | 2,75 |
| Carletti et al. (2007) | 1,000 | 0,0430 | 0,916 to 1,000 |  |  | 0,55 | 5,11 |
| Cecilia et al. (2014) | 1,000 | 0,0127 | 0,975 to 1,000 |  |  | 6,24 | 8,82 |
| Chen et al. (2015) | 0,938 | 0,0511 | 0,837 to 1,000 |  |  | 0,39 | 4,29 |
| Chiam et al. (1) (2013) | 1,000 | 0,0430 | 0,916 to 1,000 |  |  | 0,55 | 5,11 |
| Chiam et al. (2) (2013) | 1,000 | 0,0430 | 0,916 to 1,000 |  |  | 0,55 | 5,11 |
| Edwards et al. (2007) | 0,932 | 0,0440 | 0,846 to 1,000 |  |  | 0,52 | 5,01 |
| Edwards et al. (2017) | 0,989 | 0,0153 | 0,959 to 1,000 |  |  | 4,30 | 8,55 |
| Lim et al. (2023) | 1,000 | 0,181 | 0,646 to 1,000 |  |  | 0,031 | 0,59 |
| Liu et al. (2018) | 1,000 | 0,215 | 0,579 to 1,000 |  |  | 0,022 | 0,42 |
| Mishra et al. (2019) | 1,000 | 0,00423 | 0,992 to 1,000 |  |  | 56,39 | 9,40 |
| Naze et al. (2009) | 1,000 | 0,0181 | 0,964 to 1,000 |  |  | 3,07 | 8,23 |
| Pabbaraju et al. (2016) | 1,000 | 0,0630 | 0,876 to 1,000 |  |  | 0,25 | 3,34 |
| Santiago et al. (2018) | 1,000 | 0,00653 | 0,987 to 1,000 |  |  | 23,71 | 9,30 |
| Santosh et al. (2007) | 0,750 | 0,0942 | 0,565 to 0,935 |  |  | 0,11 | 1,86 |
| Simmons et al. (2016) | 1,000 | 0,0787 | 0,846 to 1,000 |  |  | 0,16 | 2,45 |
| Waggoner et al. (1) (2016) | 1,000 | 0,0364 | 0,929 to 1,000 |  |  | 0,77 | 5,88 |
| Waggoner et al. (2) (2016) | 0,708 | 0,0485 | 0,613 to 0,803 |  |  | 0,43 | 4,54 |
| Wesselman et al. (2024) | 1,000 | 0,0248 | 0,951 to 1,000 |  |  | 1,64 | 7,37 |
| Total (fixed effects) | 0,997 | 0,00318 | 0,991 to 1,000 | 313,607 | <0,001 | 100,00 | 100,00 |
| Total (random effects) | 0,975 | 0,0143 | 0,947 to 1,000 | 68,110 | <0,001 | 100,00 | 100,00 |

## Test for heterogeneity

| Q | 46,9578 |
| --- | --- |
| DF | 19 |
| Significance level | P = 0,0004 |
| I^2^ (inconsistency) | 59,54% |
| 95% CI for I^2^ | 33,85 to 75,25 |

## Publication bias

| Egger's test | |
| --- | --- |
| Intercept | -0,7937 |
| 95% CI | -1,6703 to 0,08285 |
| Significance level | P = 0,0732 |
| Begg's test | |
| Kendall's Tau | -0,6220 |
| Significance level | P = 0,0001 |

Saturday, January 11, 2025 21:10 - MedCalc^®^ version 23.1.1

# Meta-analysis: Area under ROC curve – Multiplex Specificity

| Variable for studies | Author |
| --- | --- |
| Variable for Area under ROC curve (AUC) | Especificidade |
| Variable for Standard Error of AUC | SE_Especificidade SE Especificidade |

| Study | ROC Area | Standard Error | 95% CI | z | P | Weight (%) | |
| --- | --- | --- | --- | --- | --- | --- | --- |
|  |  |  |  |  |  | Fixed | Random |
| Cecilia et al. (2014) | 1,000 | 0,0127 | 0,975 to 1,000 |  |  | 6,53 | 11,34 |
| Chen et al. (2015) | 0,938 | 0,0511 | 0,837 to 1,000 |  |  | 0,40 | 6,86 |
| Dakhave et al. (2023) | 1,000 | 0,0201 | 0,961 to 1,000 |  |  | 2,62 | 10,66 |
| Liu et al. (2018) | 1,000 | 0,215 | 0,579 to 1,000 |  |  | 0,023 | 0,86 |
| Mishra et al. (2019) | 1,000 | 0,00423 | 0,992 to 1,000 |  |  | 59,00 | 11,79 |
| Naze et al. (2009) | 1,000 | 0,0181 | 0,964 to 1,000 |  |  | 3,22 | 10,86 |
| Pabbaraju et al. (2016) | 1,000 | 0,0630 | 0,876 to 1,000 |  |  | 0,27 | 5,63 |
| Santiago et al. (2018) | 1,000 | 0,00653 | 0,987 to 1,000 |  |  | 24,81 | 11,71 |
| Simmons et al. (2016) | 1,000 | 0,0787 | 0,846 to 1,000 |  |  | 0,17 | 4,35 |
| Waggoner et al. (1) (2016) | 1,000 | 0,0364 | 0,929 to 1,000 |  |  | 0,80 | 8,66 |
| Waggoner et al. (2) (2016) | 0,708 | 0,0485 | 0,613 to 0,803 |  |  | 0,45 | 7,17 |
| Wesselman et al. (2024) | 1,000 | 0,0248 | 0,951 to 1,000 |  |  | 1,71 | 10,11 |
| Total (fixed effects) | 0,998 | 0,00325 | 0,992 to 1,000 | 306,934 | <0,001 | 100,00 | 100,00 |
| Total (random effects) | 0,975 | 0,0206 | 0,934 to 1,000 | 47,232 | <0,001 | 100,00 | 100,00 |

## Test for heterogeneity

| Q | 37,4524 |
| --- | --- |
| DF | 11 |
| Significance level | P = 0,0001 |
| I^2^ (inconsistency) | 70,63% |
| 95% CI for I^2^ | 47,03 to 83,71 |

## Publication bias

| Egger's test | |
| --- | --- |
| Intercept | -0,9261 |
| 95% CI | -2,4715 to 0,6193 |
| Significance level | P = 0,2114 |
| Begg's test | |
| Kendall's Tau | -0,7879 |
| Significance level | P = 0,0004 |

Saturday, January 11, 2025 21:13 - MedCalc^®^ version 23.1.1

# Meta-analysis: Area under ROC curve – Singleplex Specificity

| Variable for studies | Author |
| --- | --- |
| Variable for Area under ROC curve (AUC) | Especificidade |
| Variable for Standard Error of AUC | SE_Especificidade SE Especificidade |

| Study | ROC Area | Standard Error | 95% CI | z | P | Weight (%) | |
| --- | --- | --- | --- | --- | --- | --- | --- |
|  |  |  |  |  |  | Fixed | Random |
| Ali et al. (2010) | 1,000 | 0,0942 | 0,815 to 1,000 |  |  | 1,49 | 2,64 |
| Andrew et al. (1) (2023) | 1,000 | 0,0727 | 0,857 to 1,000 |  |  | 2,50 | 4,28 |
| Andrew et al. (2) (2023) | 1,000 | 0,154 | 0,699 to 1,000 |  |  | 0,56 | 1,02 |
| Carletti et al. (2007) | 1,000 | 0,0430 | 0,916 to 1,000 |  |  | 7,17 | 10,57 |
| Chiam et al. (1) (2013) | 1,000 | 0,0430 | 0,916 to 1,000 |  |  | 7,17 | 10,57 |
| Chiam et al. (2) (2013) | 1,000 | 0,0430 | 0,916 to 1,000 |  |  | 7,17 | 10,57 |
| Edwards et al. (2007) | 0,932 | 0,0440 | 0,846 to 1,000 |  |  | 6,85 | 10,19 |
| Edwards et al. (2017) | 0,989 | 0,0153 | 0,959 to 1,000 |  |  | 56,28 | 33,97 |
| Lanciotti et al. (2007) | 0,571 | 0,183 | 0,213 to 0,930 |  |  | 0,40 | 0,73 |
| Lim et al. (2023) | 1,000 | 0,181 | 0,646 to 1,000 |  |  | 0,41 | 0,75 |
| Panning et al. (2009) | 1,000 | 0,0394 | 0,923 to 1,000 |  |  | 8,53 | 12,09 |
| Santosh et al. (2007) | 0,750 | 0,0942 | 0,565 to 0,935 |  |  | 1,49 | 2,64 |
| Total (fixed effects) | 0,984 | 0,0115 | 0,961 to 1,000 | 85,522 | <0,001 | 100,00 | 100,00 |
| Total (random effects) | 0,980 | 0,0157 | 0,949 to 1,000 | 62,320 | <0,001 | 100,00 | 100,00 |

## Test for heterogeneity

| Q | 13,4363 |
| --- | --- |
| DF | 11 |
| Significance level | P = 0,2658 |
| I^2^ (inconsistency) | 18,13% |
| 95% CI for I^2^ | 0,00 to 57,35 |

## Publication bias

| Egger's test | |
| --- | --- |
| Intercept | -0,7367 |
| 95% CI | -1,8159 to 0,3425 |
| Significance level | P = 0,1592 |
| Begg's test | |
| Kendall's Tau | -0,7680 |
| Significance level | P = 0,0005 |

Saturday, January 11, 2025 21:14 - MedCalc^®^ version 23.1.1

# Meta-analysis: Area under ROC curve – Probe Specificity

| Variable for studies | Author |
| --- | --- |
| Variable for Area under ROC curve (AUC) | Especificidade |
| Variable for Standard Error of AUC | SE_Especificidade SE Especificidade |

| Study | ROC Area | Standard Error | 95% CI | z | P | Weight (%) | |
| --- | --- | --- | --- | --- | --- | --- | --- |
|  |  |  |  |  |  | Fixed | Random |
| Andrew et al. (1) (2023) | 1,000 | 0,0727 | 0,857 to 1,000 |  |  | 0,19 | 2,52 |
| Andrew et al. (2) (2023) | 1,000 | 0,154 | 0,699 to 1,000 |  |  | 0,042 | 0,69 |
| Carletti et al. (2007) | 1,000 | 0,0430 | 0,916 to 1,000 |  |  | 0,54 | 4,99 |
| Cecilia et al. (2014) | 1,000 | 0,0127 | 0,975 to 1,000 |  |  | 6,15 | 9,61 |
| Dakhave et al. (2023) | 1,000 | 0,0201 | 0,961 to 1,000 |  |  | 2,47 | 8,49 |
| Edwards et al. (2007) | 0,932 | 0,0440 | 0,846 to 1,000 |  |  | 0,52 | 4,87 |
| Edwards et al. (2017) | 0,989 | 0,0153 | 0,959 to 1,000 |  |  | 4,24 | 9,24 |
| Lanciotti et al. (2007) | 0,571 | 0,183 | 0,213 to 0,930 |  |  | 0,030 | 0,50 |
| Lim et al. (2023) | 1,000 | 0,181 | 0,646 to 1,000 |  |  | 0,031 | 0,51 |
| Liu et al. (2018) | 1,000 | 0,215 | 0,579 to 1,000 |  |  | 0,022 | 0,37 |
| Mishra et al. (2019) | 1,000 | 0,00423 | 0,992 to 1,000 |  |  | 55,55 | 10,44 |
| Naze et al. (2009) | 1,000 | 0,0181 | 0,964 to 1,000 |  |  | 3,03 | 8,80 |
| Pabbaraju et al. (2016) | 1,000 | 0,0630 | 0,876 to 1,000 |  |  | 0,25 | 3,10 |
| Panning et al. (2009) | 1,000 | 0,0394 | 0,923 to 1,000 |  |  | 0,64 | 5,45 |
| Santiago et al. (2018) | 1,000 | 0,00653 | 0,987 to 1,000 |  |  | 23,36 | 10,29 |
| Simmons et al. (2016) | 1,000 | 0,0787 | 0,846 to 1,000 |  |  | 0,16 | 2,22 |
| Waggoner et al. (1) (2016) | 1,000 | 0,0364 | 0,929 to 1,000 |  |  | 0,75 | 5,87 |
| Waggoner et al. (2) (2016) | 0,708 | 0,0485 | 0,613 to 0,803 |  |  | 0,42 | 4,36 |
| Wesselman et al. (2024) | 1,000 | 0,0248 | 0,951 to 1,000 |  |  | 1,61 | 7,69 |
| Total (fixed effects) | 0,998 | 0,00316 | 0,992 to 1,000 | 316,123 | <0,001 | 100,00 | 100,00 |
| Total (random effects) | 0,981 | 0,0132 | 0,955 to 1,000 | 74,233 | <0,001 | 100,00 | 100,00 |

## Test for heterogeneity

| Q | 44,1217 |
| --- | --- |
| DF | 18 |
| Significance level | P = 0,0006 |
| I^2^ (inconsistency) | 59,20% |
| 95% CI for I^2^ | 32,38 to 75,39 |

## Publication bias

| Egger's test | |
| --- | --- |
| Intercept | -0,7543 |
| 95% CI | -1,6749 to 0,1663 |
| Significance level | P = 0,1020 |
| Begg's test | |
| Kendall's Tau | -0,6959 |
| Significance level | P < 0,0001 |

Saturday, January 11, 2025 21:15 - MedCalc^®^ version 23.1.1

# Meta-analysis: Area under ROC curve – SYBR green Specificity

| Variable for studies | Author |
| --- | --- |
| Variable for Area under ROC curve (AUC) | Especificidade |
| Variable for Standard Error of AUC | SE_Especificidade SE Especificidade |

| Study | ROC Area | Standard Error | 95% CI | z | P | Weight (%) | |
| --- | --- | --- | --- | --- | --- | --- | --- |
|  |  |  |  |  |  | Fixed | Random |
| Ali et al. (2010) | 1,000 | 0,0942 | 0,815 to 1,000 |  |  | 6,66 | 10,40 |
| Chen et al. (2015) | 0,938 | 0,0511 | 0,837 to 1,000 |  |  | 22,62 | 23,53 |
| Chiam et al. (1) (2013) | 1,000 | 0,0430 | 0,916 to 1,000 |  |  | 32,03 | 27,83 |
| Chiam et al. (2) (2013) | 1,000 | 0,0430 | 0,916 to 1,000 |  |  | 32,03 | 27,83 |
| Santosh et al. (2007) | 0,750 | 0,0942 | 0,565 to 0,935 |  |  | 6,66 | 10,40 |
| Total (fixed effects) | 0,969 | 0,0243 | 0,921 to 1,000 | 39,860 | <0,001 | 100,00 | 100,00 |
| Total (random effects) | 0,959 | 0,0342 | 0,892 to 1,000 | 28,064 | <0,001 | 100,00 | 100,00 |

## Test for heterogeneity

| Q | 6,9228 |
| --- | --- |
| DF | 4 |
| Significance level | P = 0,1400 |
| I^2^ (inconsistency) | 42,22% |
| 95% CI for I^2^ | 0,00 to 78,74 |

## Publication bias

| Egger's test | |
| --- | --- |
| Intercept | -2,4199 |
| 95% CI | -7,7009 to 2,8611 |
| Significance level | P = 0,2408 |
| Begg's test | |
| Kendall's Tau | -0,7071 |
| Significance level | P = 0,0833 |

Saturday, January 11, 2025 21:16 - MedCalc^®^ version 23.1.1

# Meta-analysis: Area under ROC curve - One-step Sensitivity

| Variable for studies | Author |
| --- | --- |
| Variable for Area under ROC curve (AUC) | Sensibilidade |
| Variable for Standard Error of AUC | SE_Sensibilidade SE Sensibilidade |

| Study | ROC Area | Standard Error | 95% CI | z | P | Weight (%) | |
| --- | --- | --- | --- | --- | --- | --- | --- |
|  |  |  |  |  |  | Fixed | Random |
| Ali et al. (2010) | 1,000 | 0,0858 | 0,832 to 1,000 |  |  | 0,62 | 1,13 |
| Andrew et al. (1) (2023) | 0,757 | 0,0751 | 0,610 to 0,904 |  |  | 0,81 | 1,45 |
| Andrew et al. (2) (2023) | 0,414 | 0,0958 | 0,226 to 0,601 |  |  | 0,50 | 0,92 |
| Carletti et al. (2007) | 1,000 | 0,154 | 0,699 to 1,000 |  |  | 0,19 | 0,37 |
| Cecilia et al. (2014) | 0,955 | 0,0580 | 0,841 to 1,000 |  |  | 1,36 | 2,31 |
| Chen et al. (2015) | 1,000 | 0,0201 | 0,961 to 1,000 |  |  | 11,34 | 10,09 |
| Dakhave et al. (2023) | 1,000 | 0,0556 | 0,891 to 1,000 |  |  | 1,48 | 2,48 |
| Edwards et al. (2007) | 1,000 | 0,0727 | 0,857 to 1,000 |  |  | 0,87 | 1,54 |
| Edwards et al. (2017) | 0,986 | 0,0185 | 0,950 to 1,000 |  |  | 13,29 | 10,81 |
| Lim et al. (2023) | 1,000 | 0,117 | 0,770 to 1,000 |  |  | 0,33 | 0,62 |
| Liu et al. (2018) | 1,000 | 0,0942 | 0,815 to 1,000 |  |  | 0,51 | 0,95 |
| Mishra et al. (2019) | 1,000 | 0,0430 | 0,916 to 1,000 |  |  | 2,48 | 3,82 |
| Naze et al. (2009) | 1,000 | 0,0295 | 0,942 to 1,000 |  |  | 5,25 | 6,58 |
| Pabbaraju et al. (2016) | 1,000 | 0,215 | 0,579 to 1,000 |  |  | 0,099 | 0,19 |
| Panning et al. (2009) | 1,000 | 0,0255 | 0,950 to 1,000 |  |  | 7,02 | 7,87 |
| Santiago et al. (2018) | 1,000 | 0,0630 | 0,876 to 1,000 |  |  | 1,15 | 1,99 |
| Santosh et al. (2007) | 1,000 | 0,0242 | 0,952 to 1,000 |  |  | 7,80 | 8,35 |
| Simmons et al. (2016) | 1,000 | 0,0225 | 0,956 to 1,000 |  |  | 9,05 | 9,04 |
| Waggoner et al. (1) (2016) | 1,000 | 0,0160 | 0,969 to 1,000 |  |  | 17,86 | 12,12 |
| Waggoner et al. (2) (2016) | 0,904 | 0,0283 | 0,848 to 0,960 |  |  | 5,69 | 6,93 |
| Wesselman et al. (2024) | 1,000 | 0,0193 | 0,962 to 1,000 |  |  | 12,32 | 10,47 |
| Total (fixed effects) | 0,987 | 0,00676 | 0,974 to 1,000 | 146,032 | <0,001 | 100,00 | 100,00 |
| Total (random effects) | 0,982 | 0,00941 | 0,963 to 1,000 | 104,334 | <0,001 | 100,00 | 100,00 |

## Test for heterogeneity

| Q | 56,9719 |
| --- | --- |
| DF | 20 |
| Significance level | P < 0,0001 |
| I^2^ (inconsistency) | 64,89% |
| 95% CI for I^2^ | 44,21 to 77,91 |

## Publication bias

| Egger's test | |
| --- | --- |
| Intercept | -1,1072 |
| 95% CI | -2,4435 to 0,2291 |
| Significance level | P = 0,0991 |
| Begg's test | |
| Kendall's Tau | -0,6095 |
| Significance level | P = 0,0001 |

Saturday, January 11, 2025 20:42 - MedCalc^®^ version 23.1.1

# Meta-analysis: Area under ROC curve - Two-step Sensitivity

| Variable for studies | Author |
| --- | --- |
| Variable for Area under ROC curve (AUC) | Sensibilidade |
| Variable for Standard Error of AUC | SE_Sensibilidade SE Sensibilidade |

| Study | ROC Area | Standard Error | 95% CI | z | P | Weight (%) | |
| --- | --- | --- | --- | --- | --- | --- | --- |
|  |  |  |  |  |  | Fixed | Random |
| Chiam et al. (1) (2013) | 0,867 | 0,0688 | 0,732 to 1,000 |  |  | 28,78 | 35,91 |
| Chiam et al. (2) (2013) | 0,967 | 0,0437 | 0,881 to 1,000 |  |  | 71,22 | 64,09 |
| Total (fixed effects) | 0,938 | 0,0369 | 0,866 to 1,000 | 25,419 | <0,001 | 100,00 | 100,00 |
| Total (random effects) | 0,931 | 0,0480 | 0,837 to 1,000 | 19,402 | <0,001 | 100,00 | 100,00 |

## Test for heterogeneity

| Q | 1,5056 |
| --- | --- |
| DF | 1 |
| Significance level | P = 0,2198 |
| I^2^ (inconsistency) | 33,58% |
| 95% CI for I^2^ | 0,00 to 0,00 |

## Publication bias

| Egger's test | |
| --- | --- |
| Intercept | -3,9919 |
| 95% CI |  |
| Significance level | P < 0,0001 |
| Begg's test | |
| Kendall's Tau | -1,0000 |
| Significance level | P = 0,3173 |

Saturday, January 11, 2025 20:44 - MedCalc^®^ version 23.1.1

# Meta-analysis: Area under ROC curve - Commercial Sensitivity

| Variable for studies | Author |
| --- | --- |
| Variable for Area under ROC curve (AUC) | Sensibilidade |
| Variable for Standard Error of AUC | SE_Sensibilidade SE Sensibilidade |

| Study | ROC Area | Standard Error | 95% CI | z | P | Weight (%) | |
| --- | --- | --- | --- | --- | --- | --- | --- |
|  |  |  |  |  |  | Fixed | Random |
| Andrew et al. (2) (2023) | 0,414 | 0,0958 | 0,226 to 0,601 |  |  | 5,54 | 31,12 |
| Dakhave et al. (2023) | 1,000 | 0,0556 | 0,891 to 1,000 |  |  | 16,42 | 33,83 |
| Panning et al. (2009) | 1,000 | 0,0255 | 0,950 to 1,000 |  |  | 78,04 | 35,05 |
| Total (fixed effects) | 0,967 | 0,0225 | 0,923 to 1,000 | 42,929 | <0,001 | 100,00 | 100,00 |
| Total (random effects) | 0,817 | 0,154 | 0,516 to 1,000 | 5,312 | <0,001 | 100,00 | 100,00 |

## Test for heterogeneity

| Q | 35,3822 |
| --- | --- |
| DF | 2 |
| Significance level | P < 0,0001 |
| I^2^ (inconsistency) | 94,35% |
| 95% CI for I^2^ | 86,87 to 97,57 |

## Publication bias

| Egger's test | |
| --- | --- |
| Intercept | -5,7936 |
| 95% CI | -60,6764 to 49,0893 |
| Significance level | P = 0,4078 |
| Begg's test | |
| Kendall's Tau | -1,0000 |
| Significance level | P = 0,1172 |

Saturday, January 11, 2025 20:38 - MedCalc^®^ version 23.1.1

# Meta-analysis: Area under ROC curve - in-house Sensitivity

| Variable for studies | Author |
| --- | --- |
| Variable for Area under ROC curve (AUC) | Sensibilidade |
| Variable for Standard Error of AUC | SE_Sensibilidade SE Sensibilidade |

| Study | ROC Area | Standard Error | 95% CI | z | P | Weight (%) | |
| --- | --- | --- | --- | --- | --- | --- | --- |
|  |  |  |  |  |  | Fixed | Random |
| Ali et al. (2010) | 1,000 | 0,0858 | 0,832 to 1,000 |  |  | 0,66 | 1,00 |
| Andrew et al. (1) (2023) | 0,757 | 0,0751 | 0,610 to 0,904 |  |  | 0,86 | 1,29 |
| Carletti et al. (2007) | 1,000 | 0,154 | 0,699 to 1,000 |  |  | 0,21 | 0,32 |
| Cecilia et al. (2014) | 0,955 | 0,0580 | 0,841 to 1,000 |  |  | 1,44 | 2,10 |
| Chen et al. (2015) | 1,000 | 0,0201 | 0,961 to 1,000 |  |  | 12,01 | 11,22 |
| Chiam et al. (1) (2013) | 0,867 | 0,0688 | 0,732 to 1,000 |  |  | 1,02 | 1,53 |
| Chiam et al. (2) (2013) | 0,967 | 0,0437 | 0,881 to 1,000 |  |  | 2,53 | 3,49 |
| Edwards et al. (2007) | 1,000 | 0,0727 | 0,857 to 1,000 |  |  | 0,92 | 1,38 |
| Edwards et al. (2017) | 0,986 | 0,0185 | 0,950 to 1,000 |  |  | 14,08 | 12,29 |
| Lim et al. (2023) | 1,000 | 0,117 | 0,770 to 1,000 |  |  | 0,35 | 0,55 |
| Liu et al. (2018) | 1,000 | 0,0942 | 0,815 to 1,000 |  |  | 0,55 | 0,84 |
| Mishra et al. (2019) | 1,000 | 0,0430 | 0,916 to 1,000 |  |  | 2,62 | 3,60 |
| Naze et al. (2009) | 1,000 | 0,0295 | 0,942 to 1,000 |  |  | 5,56 | 6,65 |
| Pabbaraju et al. (2016) | 1,000 | 0,215 | 0,579 to 1,000 |  |  | 0,10 | 0,16 |
| Santiago et al. (2018) | 1,000 | 0,0630 | 0,876 to 1,000 |  |  | 1,22 | 1,80 |
| Santosh et al. (2007) | 1,000 | 0,0242 | 0,952 to 1,000 |  |  | 8,26 | 8,85 |
| Simmons et al. (2016) | 1,000 | 0,0225 | 0,956 to 1,000 |  |  | 9,59 | 9,76 |
| Waggoner et al. (1) (2016) | 1,000 | 0,0160 | 0,969 to 1,000 |  |  | 18,93 | 14,31 |
| Waggoner et al. (2) (2016) | 0,904 | 0,0283 | 0,848 to 0,960 |  |  | 6,03 | 7,07 |
| Wesselman et al. (2024) | 1,000 | 0,0193 | 0,962 to 1,000 |  |  | 13,05 | 11,78 |
| Total (fixed effects) | 0,987 | 0,00696 | 0,974 to 1,000 | 141,872 | <0,001 | 100,00 | 100,00 |
| Total (random effects) | 0,984 | 0,00875 | 0,967 to 1,000 | 112,516 | <0,001 | 100,00 | 100,00 |

## Test for heterogeneity

| Q | 24,1106 |
| --- | --- |
| DF | 19 |
| Significance level | P = 0,1919 |
| I^2^ (inconsistency) | 21,20% |
| 95% CI for I^2^ | 0,00 to 54,17 |

## Publication bias

| Egger's test | |
| --- | --- |
| Intercept | -0,6672 |
| 95% CI | -1,5956 to 0,2613 |
| Significance level | P = 0,1485 |
| Begg's test | |
| Kendall's Tau | -0,4737 |
| Significance level | P = 0,0035 |

Saturday, January 11, 2025 20:41 - MedCalc^®^ version 23.1.1

# Meta-analysis: Area under ROC curve multiplex Sensitivity

| Variable for studies | Author |
| --- | --- |
| Variable for Area under ROC curve (AUC) | Sensibilidade |
| Variable for Standard Error of AUC | SE_Sensibilidade SE Sensibilidade |

| Study | ROC Area | Standard Error | 95% CI | z | P | Weight (%) | |
| --- | --- | --- | --- | --- | --- | --- | --- |
|  |  |  |  |  |  | Fixed | Random |
| Cecilia et al. (2014) | 0,955 | 0,0580 | 0,841 to 1,000 |  |  | 1,98 | 1,98 |
| Chen et al. (2015) | 1,000 | 0,0201 | 0,961 to 1,000 |  |  | 16,53 | 16,53 |
| Dakhave et al. (2023) | 1,000 | 0,0556 | 0,891 to 1,000 |  |  | 2,15 | 2,15 |
| Liu et al. (2018) | 1,000 | 0,0942 | 0,815 to 1,000 |  |  | 0,75 | 0,75 |
| Mishra et al. (2019) | 1,000 | 0,0430 | 0,916 to 1,000 |  |  | 3,61 | 3,61 |
| Naze et al. (2009) | 1,000 | 0,0295 | 0,942 to 1,000 |  |  | 7,65 | 7,65 |
| Pabbaraju et al. (2016) | 1,000 | 0,215 | 0,579 to 1,000 |  |  | 0,14 | 0,14 |
| Santiago et al. (2018) | 1,000 | 0,0630 | 0,876 to 1,000 |  |  | 1,68 | 1,68 |
| Simmons et al. (2016) | 1,000 | 0,0225 | 0,956 to 1,000 |  |  | 13,19 | 13,19 |
| Waggoner et al. (1) (2016) | 1,000 | 0,0160 | 0,969 to 1,000 |  |  | 26,05 | 26,05 |
| Waggoner et al. (2) (2016) | 0,904 | 0,0283 | 0,848 to 0,960 |  |  | 8,30 | 8,30 |
| Wesselman et al. (2024) | 1,000 | 0,0193 | 0,962 to 1,000 |  |  | 17,96 | 17,96 |
| Total (fixed effects) | 0,991 | 0,00816 | 0,975 to 1,000 | 121,406 | <0,001 | 100,00 | 100,00 |
| Total (random effects) | 0,991 | 0,00816 | 0,975 to 1,000 | 121,406 | <0,001 | 100,00 | 100,00 |

## Test for heterogeneity

| Q | 10,8859 |
| --- | --- |
| DF | 11 |
| Significance level | P = 0,4529 |
| I^2^ (inconsistency) | 0,00% |
| 95% CI for I^2^ | 0,00 to 58,00 |

## Publication bias

| Egger's test | |
| --- | --- |
| Intercept | -0,3042 |
| 95% CI | -1,6297 to 1,0213 |
| Significance level | P = 0,6202 |
| Begg's test | |
| Kendall's Tau | -0,6970 |
| Significance level | P = 0,0016 |

Saturday, January 11, 2025 20:47 - MedCalc^®^ version 23.1.1

# Meta-analysis: Area under ROC curve singleplex Sensitivity

| Variable for studies | Author |
| --- | --- |
| Variable for Area under ROC curve (AUC) | Sensibilidade |
| Variable for Standard Error of AUC | SE_Sensibilidade SE Sensibilidade |

| Study | ROC Area | Standard Error | 95% CI | z | P | Weight (%) | |
| --- | --- | --- | --- | --- | --- | --- | --- |
|  |  |  |  |  |  | Fixed | Random |
| Ali et al. (2010) | 1,000 | 0,0858 | 0,832 to 1,000 |  |  | 1,77 | 7,85 |
| Andrew et al. (1) (2023) | 0,757 | 0,0751 | 0,610 to 0,904 |  |  | 2,31 | 8,43 |
| Andrew et al. (2) (2023) | 0,414 | 0,0958 | 0,226 to 0,601 |  |  | 1,42 | 7,32 |
| Carletti et al. (2007) | 1,000 | 0,154 | 0,699 to 1,000 |  |  | 0,55 | 4,75 |
| Chiam et al. (1) (2013) | 0,867 | 0,0688 | 0,732 to 1,000 |  |  | 2,76 | 8,78 |
| Chiam et al. (2) (2013) | 0,967 | 0,0437 | 0,881 to 1,000 |  |  | 6,82 | 10,05 |
| Edwards et al. (2007) | 1,000 | 0,0727 | 0,857 to 1,000 |  |  | 2,47 | 8,56 |
| Edwards et al. (2017) | 0,986 | 0,0185 | 0,950 to 1,000 |  |  | 37,92 | 10,94 |
| Lanciotti et al. (2007) | 1,000 | 0,133 | 0,739 to 1,000 |  |  | 0,74 | 5,54 |
| Lim et al. (2023) | 1,000 | 0,117 | 0,770 to 1,000 |  |  | 0,95 | 6,25 |
| Panning et al. (2009) | 1,000 | 0,0255 | 0,950 to 1,000 |  |  | 20,04 | 10,75 |
| Santosh et al. (2007) | 1,000 | 0,0242 | 0,952 to 1,000 |  |  | 22,25 | 10,79 |
| Total (fixed effects) | 0,975 | 0,0114 | 0,953 to 0,997 | 85,371 | <0,001 | 100,00 | 100,00 |
| Total (random effects) | 0,920 | 0,0442 | 0,833 to 1,000 | 20,831 | <0,001 | 100,00 | 100,00 |

## Test for heterogeneity

| Q | 48,0072 |
| --- | --- |
| DF | 11 |
| Significance level | P < 0,0001 |
| I^2^ (inconsistency) | 77,09% |
| 95% CI for I^2^ | 60,14 to 86,83 |

## Publication bias

| Egger's test | |
| --- | --- |
| Intercept | -1,5700 |
| 95% CI | -3,6597 to 0,5197 |
| Significance level | P = 0,1251 |
| Begg's test | |
| Kendall's Tau | -0,4242 |
| Significance level | P = 0,0549 |

Saturday, January 11, 2025 20:48 - MedCalc^®^ version 23.1.1

# Meta-analysis: Area under ROC curve – Probe Sensitivity

| Variable for studies | Author |
| --- | --- |
| Variable for Area under ROC curve (AUC) | Sensibilidade |
| Variable for Standard Error of AUC | SE_Sensibilidade SE Sensibilidade |

| Study | ROC Area | Standard Error | 95% CI | z | P | Weight (%) | |
| --- | --- | --- | --- | --- | --- | --- | --- |
|  |  |  |  |  |  | Fixed | Random |
| Andrew et al. (1) (2023) | 0,757 | 0,0751 | 0,610 to 0,904 |  |  | 1,01 | 4,36 |
| Andrew et al. (2) (2023) | 0,414 | 0,0958 | 0,226 to 0,601 |  |  | 0,62 | 3,35 |
| Carletti et al. (2007) | 1,000 | 0,154 | 0,699 to 1,000 |  |  | 0,24 | 1,72 |
| Cecilia et al. (2014) | 0,955 | 0,0580 | 0,841 to 1,000 |  |  | 1,69 | 5,43 |
| Dakhave et al. (2023) | 1,000 | 0,0556 | 0,891 to 1,000 |  |  | 1,84 | 5,59 |
| Edwards et al. (2007) | 1,000 | 0,0727 | 0,857 to 1,000 |  |  | 1,07 | 4,50 |
| Edwards et al. (2017) | 0,986 | 0,0185 | 0,950 to 1,000 |  |  | 16,50 | 8,00 |
| Lanciotti et al. (2007) | 1,000 | 0,133 | 0,739 to 1,000 |  |  | 0,32 | 2,14 |
| Lim et al. (2023) | 1,000 | 0,117 | 0,770 to 1,000 |  |  | 0,41 | 2,57 |
| Liu et al. (2018) | 1,000 | 0,0942 | 0,815 to 1,000 |  |  | 0,64 | 3,41 |
| Mishra et al. (2019) | 1,000 | 0,0430 | 0,916 to 1,000 |  |  | 3,08 | 6,47 |
| Naze et al. (2009) | 1,000 | 0,0295 | 0,942 to 1,000 |  |  | 6,52 | 7,39 |
| Pabbaraju et al. (2016) | 1,000 | 0,215 | 0,579 to 1,000 |  |  | 0,12 | 0,97 |
| Panning et al. (2009) | 1,000 | 0,0255 | 0,950 to 1,000 |  |  | 8,72 | 7,64 |
| Santiago et al. (2018) | 1,000 | 0,0630 | 0,876 to 1,000 |  |  | 1,43 | 5,09 |
| Simmons et al. (2016) | 1,000 | 0,0225 | 0,956 to 1,000 |  |  | 11,24 | 7,81 |
| Waggoner et al. (1) (2016) | 1,000 | 0,0160 | 0,969 to 1,000 |  |  | 22,19 | 8,12 |
| Waggoner et al. (2) (2016) | 0,904 | 0,0283 | 0,848 to 0,960 |  |  | 7,07 | 7,46 |
| Wesselman et al. (2024) | 1,000 | 0,0193 | 0,962 to 1,000 |  |  | 15,30 | 7,97 |
| Total (fixed effects) | 0,984 | 0,00753 | 0,969 to 0,999 | 130,616 | <0,001 | 100,00 | 100,00 |
| Total (random effects) | 0,959 | 0,0225 | 0,915 to 1,000 | 42,544 | <0,001 | 100,00 | 100,00 |

## Test for heterogeneity

| Q | 56,1110 |
| --- | --- |
| DF | 18 |
| Significance level | P < 0,0001 |
| I^2^ (inconsistency) | 67,92% |
| 95% CI for I^2^ | 48,34 to 80,08 |

## Publication bias

| Egger's test | |
| --- | --- |
| Intercept | -1,0595 |
| 95% CI | -2,4729 to 0,3540 |
| Significance level | P = 0,1322 |
| Begg's test | |
| Kendall's Tau | -0,5789 |
| Significance level | P = 0,0005 |

Saturday, January 11, 2025 20:51 - MedCalc^®^ version 23.1.1

# Meta-analysis: Area under ROC curve - SYBR green Sensitivity

| Variable for studies | Author |
| --- | --- |
| Variable for Area under ROC curve (AUC) | Sensibilidade |
| Variable for Standard Error of AUC | SE_Sensibilidade SE Sensibilidade |

| Study | ROC Area | Standard Error | 95% CI | z | P | Weight (%) | |
| --- | --- | --- | --- | --- | --- | --- | --- |
|  |  |  |  |  |  | Fixed | Random |
| Ali et al. (2010) | 1,000 | 0,0858 | 0,832 to 1,000 |  |  | 2,69 | 2,69 |
| Chen et al. (2015) | 1,000 | 0,0201 | 0,961 to 1,000 |  |  | 49,05 | 49,05 |
| Chiam et al. (1) (2013) | 0,867 | 0,0688 | 0,732 to 1,000 |  |  | 4,18 | 4,18 |
| Chiam et al. (2) (2013) | 0,967 | 0,0437 | 0,881 to 1,000 |  |  | 10,34 | 10,34 |
| Santosh et al. (2007) | 1,000 | 0,0242 | 0,952 to 1,000 |  |  | 33,74 | 33,74 |
| Total (fixed effects) | 0,991 | 0,0141 | 0,963 to 1,000 | 70,469 | <0,001 | 100,00 | 100,00 |
| Total (random effects) | 0,991 | 0,0141 | 0,963 to 1,000 | 70,469 | <0,001 | 100,00 | 100,00 |

## Test for heterogeneity

| Q | 3,9177 |
| --- | --- |
| DF | 4 |
| Significance level | P = 0,4173 |
| I^2^ (inconsistency) | 0,00% |
| 95% CI for I^2^ | 0,00 to 80,01 |

## Publication bias

| Egger's test | |
| --- | --- |
| Intercept | -1,2869 |
| 95% CI | -3,7922 to 1,2185 |
| Significance level | P = 0,2006 |
| Begg's test | |
| Kendall's Tau | -0,6000 |
| Significance level | P = 0,1416 |

Saturday, January 11, 2025 20:54 - MedCalc^®^ version 23.1.1

# Meta-analysis: Area under ROC curve – High and Unclear risk

| Variable for studies | Reference |
| --- | --- |
| Variable for Area under ROC curve (AUC) | Accuracy |
| Variable for Standard Error of AUC | SE |

| Study | ROC Area | Standard Error | 95% CI | z | P | Weight (%) | |
| --- | --- | --- | --- | --- | --- | --- | --- |
|  |  |  |  |  |  | Fixed | Random |
| Santosh et al. (2007) | 0,902 | 0,0421 | 0,819 to 0,984 |  |  | 0,55 | 5,29 |
| Edwards et al. (2007) | 0,946 | 0,0357 | 0,876 to 1,000 |  |  | 0,77 | 6,00 |
| Lanciotti et al. (2007) | 0,750 | 0,132 | 0,492 to 1,000 |  |  | 0,056 | 1,12 |
| Carletti et al. (2007) | 1,000 | 0,0364 | 0,929 to 1,000 |  |  | 0,74 | 5,92 |
| Panning et al. (2009) | 1,000 | 0,0160 | 0,969 to 1,000 |  |  | 3,83 | 8,28 |
| Naze et al. (2009) | 1,000 | 0,0115 | 0,977 to 1,000 |  |  | 7,40 | 8,68 |
| Ali et al. (2010) | 1,000 | 0,0498 | 0,902 to 1,000 |  |  | 0,40 | 4,52 |
| Chiam et al. (1) (2013) | 0,920 | 0,0434 | 0,835 to 1,000 |  |  | 0,52 | 5,15 |
| Chiam et al. (2) (2013) | 0,980 | 0,0260 | 0,929 to 1,000 |  |  | 1,45 | 7,15 |
| Cecilia et al. (2014) | 0,989 | 0,0147 | 0,961 to 1,000 |  |  | 4,54 | 8,40 |
| Chen et al. (2015) | 0,947 | 0,0223 | 0,903 to 0,991 |  |  | 1,97 | 7,59 |
| Mishra et al. (2019) | 1,000 | 0,00388 | 0,992 to 1,000 |  |  | 65,19 | 9,10 |
| Andrew et al. (1) (2023) | 0,813 | 0,0604 | 0,694 to 0,931 |  |  | 0,27 | 3,65 |
| Andrew et al. (2) (2023) | 0,485 | 0,0910 | 0,306 to 0,663 |  |  | 0,12 | 2,07 |
| Dakhave et al. (2023) | 1,000 | 0,0152 | 0,970 to 1,000 |  |  | 4,24 | 8,36 |
| Wesselman et al. (2024) | 1,000 | 0,0111 | 0,978 to 1,000 |  |  | 7,95 | 8,71 |
| Total (fixed effects) | 0,995 | 0,00313 | 0,989 to 1,000 | 317,969 | <0,001 | 100,00 | 100,00 |
| Total (random effects) | 0,961 | 0,0149 | 0,932 to 0,990 | 64,526 | <0,001 | 100,00 | 100,00 |

## Test for heterogeneity

| Q | 61,1555 |
| --- | --- |
| DF | 15 |
| Significance level | P < 0,0001 |
| I^2^ (inconsistency) | 75,47% |
| 95% CI for I^2^ | 60,15 to 84,91 |

## Publication bias

| Egger's test | |
| --- | --- |
| Intercept | -1,9121 |
| 95% CI | -2,9845 to -0,8398 |
| Significance level | P = 0,0019 |
| Begg's test | |
| Kendall's Tau | -0,6667 |
| Significance level | P = 0,0003 |

Wednesday, January 28, 2026 10:33 - MedCalc^®^ version 23.4.8

# Meta-analysis: Area under ROC curve – Low risk

| Variable for studies | Reference |
| --- | --- |
| Variable for Area under ROC curve (AUC) | Accuracy |
| Variable for Standard Error of AUC | SE |

| Study | ROC Area | Standard Error | 95% CI | z | P | Weight (%) | |
| --- | --- | --- | --- | --- | --- | --- | --- |
|  |  |  |  |  |  | Fixed | Random |
| Waggoner et al. (1) (2016) | 1,000 | 0,0114 | 0,978 to 1,000 |  |  | 15,69 | 17,20 |
| Simmons et al. (2016) | 1,000 | 0,0181 | 0,964 to 1,000 |  |  | 6,15 | 15,95 |
| Pabbaraju et al. (2016) | 1,000 | 0,0556 | 0,891 to 1,000 |  |  | 0,65 | 7,97 |
| Waggoner et al. (2) (2016) | 0,838 | 0,0262 | 0,787 to 0,889 |  |  | 2,95 | 14,13 |
| Edwards et al. (2017) | 0,988 | 0,0107 | 0,967 to 1,000 |  |  | 17,70 | 17,30 |
| Liu et al. (2018) | 1,000 | 0,0787 | 0,846 to 1,000 |  |  | 0,33 | 5,11 |
| Santiago et al. (2018) | 1,000 | 0,00599 | 0,988 to 1,000 |  |  | 56,26 | 17,85 |
| Lim et al. (2023) | 1,000 | 0,0858 | 0,832 to 1,000 |  |  | 0,27 | 4,50 |
| Total (fixed effects) | 0,993 | 0,00450 | 0,984 to 1,000 | 220,830 | <0,001 | 100,00 | 100,00 |
| Total (random effects) | 0,975 | 0,0210 | 0,934 to 1,000 | 46,462 | <0,001 | 100,00 | 100,00 |

## Test for heterogeneity

| Q | 37,1755 |
| --- | --- |
| DF | 7 |
| Significance level | P < 0,0001 |
| I^2^ (inconsistency) | 81,17% |
| 95% CI for I^2^ | 63,85 to 90,19 |

## Publication bias

| Egger's test | |
| --- | --- |
| Intercept | -1,2167 |
| 95% CI | -4,3631 to 1,9296 |
| Significance level | P = 0,3806 |
| Begg's test | |
| Kendall's Tau | -0,4286 |
| Significance level | P = 0,1376 |

Wednesday, January 28, 2026 10:28 - MedCalc^®^ version 23.4.8
